# Diversification of the kinetic properties of yeast NADP‐glutamate‐dehydrogenase isozymes proceeds independently of their evolutionary origin

**DOI:** 10.1002/mbo3.419

**Published:** 2016-11-19

**Authors:** Carlos Campero‐Basaldua, Héctor Quezada, Lina Riego‐Ruíz, Dariel Márquez, Erendira Rojas, James González, Mohammed El‐Hafidi, Alicia González

**Affiliations:** ^1^ Departamento de Bioquímica y Biología EstructuralInstituto de Fisiología CelularUniversidad Nacional Autónoma de MéxicoMexico CityMéxico; ^2^ Laboratorio de Inmunología y ProteómicaHospital Infantil de México Federico GómezMexico CityMéxico; ^3^ División de Biología MolecularIPICYTSan Luis PotosíMéxico; ^4^ Departamento de Biomedicina CardiovascularInstituto Nacional de Cardiología Ignacio ChávezMexico CityMéxico

**Keywords:** functional diversification, glutamate dehydrogenase, kinetics, paralogous enzymes, phylogeny, yeast gene duplication

## Abstract

In the yeast *Saccharomyces cerevisiae*, the *ScGDH1* and *ScGDH3* encoded glutamate dehydrogenases (NADP‐GDHs) catalyze the synthesis of glutamate from ammonium and α‐ketoglutarate (α‐KG). Previous kinetic characterization showed that these enzymes displayed different allosteric properties and respectively high or low rate of α‐KG utilization. Accordingly, the coordinated action of *Sc*Gdh1 and *Sc*Gdh3, regulated balanced α‐KG utilization for glutamate biosynthesis under either fermentative or respiratory conditions, safeguarding energy provision. Here, we have addressed the question of whether there is a correlation between the regulation and kinetic properties of the NADP‐GDH isozymes present in *S. cerevisiae* (*Sc*Gdh1 and *Sc*Gdh3), *Kluyveromyces lactis* (*Kl*Gdh1), and *Lachancea kluyveri* (*Lk*Gdh1) and their evolutionary history. Our results show that the kinetic properties of *K. lactis* and *L. kluyveri* single NADP‐GDHs are respectively similar to either *ScGDH3* or *ScGDH1*, which arose from the whole genome duplication event of the *S. cerevisiae* lineage, although, *KlGDH1* and *LkGDH1* originated from a *GDH* clade, through an ancient interspecies hybridization event that preceded the divergence between the *Saccharomyces* clade and the one containing the genera *Kluyveromyces*,* Lachancea*, and *Eremothecium*. Thus, the kinetic properties which determine the NADP‐GDHs capacity to utilize α‐KG and synthesize glutamate do not correlate with their evolutionary origin.

## 1 Introduction

Two pathways determine glutamate biosynthesis in fungi: the NADP‐dependent glutamate dehydrogenase (NADP‐GDH) and the concerted action of glutamine synthetase (GS) and glutamate synthase (GOGAT) (Magasanik, 2003). These enzymes assimilate ammonium into glutamate and glutamine, whose amino groups are subsequently distributed to other compounds. The five‐carbon skeleton of these amino acids derives from α‐ketoglutarate (α‐KG), an intermediate of the tricarboxylic acid cycle. Thus, glutamate biosynthesis represents a crucial intersection of carbon and nitrogen metabolism and, as such, its regulation must balance biosynthetic needs and energy production (DeLuna, Avendano, Riego, & Gonzalez, 2001). Redox homeostasis and defense against oxidative stress are also influenced by glutamate biosynthesis since this amino acid is a glutathione precursor (Guillamon, van Riel, Giuseppin, & Verrips, 2001; Lee, Kim, Kang, Kim, & Maeng, 2012).

Although the relative contribution of the two glutamate‐producing pathways to the biosynthesis of this amino acid varies among species and growth conditions, it has been reported that NADP‐GDH (1.4.1.4) plays a leading role in *Schizosaccharomyces pombe, Aspergillus nidulans, Neurospora crassa*, and *Saccharomyces cerevisiae* grown on ammonium as sole nitrogen source, in which inactivation of the NADP‐GDH encoding genes, dramatically reduces growth rate (Fincham, 1951; Macheda, Hynes, & Davis, 1999; Magasanik, 2003; Perysinakis, Kinghorn, & Drainas, 1994). Modulation of NADP‐GDH activity in vivo coordinates metabolic fluxes according to modifications in the availability of nitrogen and carbon sources and contributes to the maintenance of an equilibrated redox state. In *Candida albicans*,* S. pombe*, and several *Aspergillus* species, this modulation involves rate of α‐ ketoglutarate (KG) utilization through allosteric regulation and cooperative kinetics (Holmes, Collings, Farnden, & Shepherd, 1989; Noor & Punekar, 2005; Perysinakis et al., 1994), whereas in *Candida tropicalis*,* Candida pseudotropicalis*,* Candida parapsilosis*,* Debaryomyces hansenii*, and *Aspergillus terreus,* NADP‐GDH hyperbolic kinetics determines intermediate utilization (Alba‐Lois et al., 2004; Choudhury & Punekar, 2009; Holmes et al., 1989).

*S. cerevisiae* has two NADP‐GDH isoforms namely *Sc*Gdh1 and *Sc*Gdh3 (Avendano, Deluna, Olivera, Valenzuela, & Gonzalez, 1997). *Sc*Gdh1 shows hyperbolic kinetics for α‐KG saturation and is the predominant isoform under exponential growth on glucose and when acetate plus raffinose are used as carbon sources (DeLuna et al., 2001; Tang, Sieg, & Trotter, 2011). While *Sc*Gdh3 is a cooperative enzyme displaying sigmoidal kinetics for α‐KG utilization, this isoform contributes significantly to NADP‐GDH activity during growth on ethanol as sole carbon source (Avendano et al., 2005; DeLuna et al., 2001) and becomes the predominant isoform during stationary phase (Lee et al., 2012). Accordingly, transcription of the *ScGDH3* gene is strongly induced during growth on ethanol and is nearly absent on glucose. This carbon‐mediated regulation is overimposed to the transcriptional activation by low nitrogen availability (Avendano et al., 2005). Although transcription of the *ScGDH1* gene is not repressed on ethanol, the relative contribution of the *Sc*Gdh1 enzyme to the overall NADP‐GDH activity is much lower than that of *Sc*Gdh3 under this condition (DeLuna et al., 2001; Riego, Avendano, DeLuna, Rodríguez, & González, 2002). It is worth mentioning that the NADP‐GDHs are not involved in glutamate catabolism, instead, the NAD‐dependent glutamate dehydrogenase (1.4.1.2) catalyzes the deamination of glutamate to ammonium and α‐KG in yeast (Miller & Magasanik, 1990).

It has been proposed that *Sc*Gdh1 and *Sc*Gdh3 kinetic differences control α‐KG utilization for biosynthetic purposes without compromising flux trough the tricarboxylic acid cycle for energy production during growth on ethanol as sole carbon source (DeLuna et al., 2001). The non‐redundant roles of *Sc*Gdh1 and *Sc*Gdh3 may be the result of an evolutionary process in which duplication of an ancestral gene and divergence of the resulting paralogous led to specialization in glutamate production under different conditions associated with the peculiar facultative metabolism of *S. cerevisiae* (Avendano et al., 2005).

It has been proposed that in the *S. cerevisiae* lineage, a whole genome duplication (WGD) event took place (Wolfe & Shields, 1997) and that a selected group of the resulting duplicated genes have been retained in two copies among which are the paralogous *ScGDH1* and *ScGDH3* genes (Seoighe & Wolfe, 1999). However, the evolutionary studies of the fungal NADP‐GDHs have not addressed the characteristics of the pre‐WGD ancestral‐type genes which did not originate through WGD, and those present in the Saccharomycetes, which arose through WGD. The *Saccharomycetales* (or *Hemyascomycetes*) group includes species closely related to *S. cerevisiae* for which the genome sequence and genetic manipulation resources are available, representing a valuable tool for functional evolutionary studies. The yeasts *Kluyveromyces lactis* and *Lachancea kluyveri* descend from the pre‐WGD ancestor, and have a single NADP‐GDH‐encoding gene, suggesting that no sporadic duplications have occurred in this gene. With regard to the carbon metabolism operating in these yeasts, it is evident that each one shows different levels of adaptation to the fermentative lifestyle: *K. lactis* metabolism is constitutively respiratory, for this reason, it cannot grow anaerobically and does not produce respiratory‐deficient mutants (Breunig et al., 2000). *L. kluyveri* displays an intermediate fermentative capacity between *K. lactis* and *S. cerevisiae,* it can grow anaerobically and produce respiratory‐deficient mutants on sugar‐rich media, but it only ferments in the absence of oxygen (Moller, Olsson, & Piskur, 2001; Moller et al., 2002), whereas in *S. cerevisiae* fermentative metabolism predominates whenever high sugar concentration is available regardless of oxygen disponibility. It even represses respiratory metabolism in the presence of high glucose or fructose concentration, through carbon catabolite repression (Gancedo, 1998). This yeast can grow anaerobically and produce respiratory‐deficient mutants (Gancedo, 1998). One of the most prominent features of baker′s yeast is the rapid conversion of sugars to ethanol and carbon dioxide under both anaerobic an aerobic conditions; this phenomenon is called Crabtree effect (Hagman, Säll, & Piskur, 2014) and is present in yeast species well adapted to the fermentative life style (Pfeiffer & Morley,^2014^). According to this classification, *S. cerevisiae* and *L. kluyveri* are Crabtree positive, whereas *K. lactis* is Crabtree negative.

This work addresses the question of whether the evolutionary origin of *S. cerevisiae Sc*Gdh1 and *Sc*Gdh3 NADP‐GDH and their corresponding orthologs in *K. lactis* and *L. kluyveri* has influenced their kinetic and transcriptional regulation. Our results show that such regulation does not correlate with the evolutionary origin of the corresponding genes, confirming that gene duplication and further functional diversification play a key role in metabolic remodeling and evolution, regardless of the origin of paralogous gene pair.

## 2 Experimental Procedures

### 2.1 Strains

Table 1 describes the characteristics of the strains used in the present work. All strains constructed for this study were derivatives of *CLA1* (*ura3 leu2*), *Lk156‐1* (*ura3*) or *KlWM37‐1* (*his3 ura3*) for *S. cerevisiae, L. kluyveri* and *K. lactis*, respectively. Mutants in *Scgdh1*Δ*::kanMx4* (*CLA2*), *Scgdh3*Δ*::LEU2* (*CLA3*), *Scglt1*Δ*::URA3* (*CLA4*), *Scgdh1*Δ*::kanMx4 Scgdh3*Δ*::LEU2* (*CLA5*), *Scgdh1*Δ*::kanMx4 Scgdh3*Δ*::LEU2 Scglt1*Δ*::URA3* (*CLA6*) and *Klglt1*Δ*::kanMx4* (*KlWM37‐3*) have been previously described (Avendano et al., 1997; DeLuna et al., 2001; Valenzuela et al., 1995).

**Table 1:** *S. cerevisiae*,* L. kluyveri*, and *K. lactis* strains used in this work

Strain	Relevant genotype	Source
*CLA1*	*MATa ScGDH1 ScGDH3 ScGLT1 ura3 leu2*	Avendano et al., 1997
*CLA2*	*MATa Scgdh1*Δ::*kanMx4 ScGDH3 ScGLT1 ura3 leu2*	DeLuna et al., 2001
*CLA3*	*MATa ScGDH1 Scgdh3*Δ::*LEU2 ScGLT1 ura3*	Avendano et al., 1997
*CLA4*	*MATa ScGDH1 ScGDH3 Scglt1*Δ::*URA3 leu2*	Avendano et al., 1997
*CLA5*	*MATa Scgdh1*Δ::*kanMx4 Scgdh3*Δ::*LEU2 ScGLT1 ura3*	DeLuna et al., 2001
*CLA6*	*MATa Scgdh1*Δ::*kanMx4 Scgdh3*Δ::*LEU2 Scglt1*Δ::*URA3*	DeLuna et al., 2001
*CLA1‐1*	*CLA1/*pRS416 *leu2*	This study
*CLA2‐1*	*CLA2/*pRS416 *leu2*	This study
*CLA2‐2*	*CLA2/*pRS416*‐ScGDH1 leu2*	This study
*CLA2‐3*	*CLA2/*pRS416*‐ScGDH3 leu2*	This study
*CLA2‐4*	*CLA2/*pRS416*‐LkGDH1 leu2*	This study
*CLA2‐5*	*CLA2/*pRS416*‐KlGDH1 leu2*	This study
*CLA5‐1*	*CLA5/*pRS416	This study
*CLA5‐2*	*CLA5/*pRS416*‐ScGDH1*	This study
*CLA5‐3*	*CLA5/*pRS416*‐ScGDH3*	This study
*CLA5‐4*	*CLA5/*pRS416*‐LkGDH1*	This study
*CLA5‐5*	*CLA5/*pRS416*‐KlGDH1*	This study
*Lk156‐1*	*Mata LkGDH1 LkGLT1 ura3*	Montalvo, J. et al. 2015
*Lk156‐2*	*Mata Lkgdh1*Δ::*kanMx4 LkGLT1 ura3*	This study
*Lk156‐3*	*Mata LkGDH1 Lkglt1*Δ*::natMx4 ura3*	This study
*Lk156‐4*	*Mata Lkgdh1*Δ::*kanMx4 Lkglt1*Δ*::natMx4 ura3*	This study
*Lk156‐1‐1*	*Lk156‐1/*p*Lk‐*EE	This study
*Lk156‐2‐1*	*Lk156‐2/*p*Lk‐*EE	This study
*Lk156‐2‐2*	*Lk156‐2/*p*Lk‐*EE *‐ScGDH1*	This study
*Lk156‐2‐3*	*Lk156‐2/*p*Lk‐*EE *‐ScGDH3*	This study
*Lk156‐2‐4*	*Lk156‐2/*p*Lk‐*EE *‐LkGDH1*	This study
*Lk156‐2‐5*	*Lk156‐2/*p*Lk‐*EE *‐KlGDH1*	This study
*KlWM37‐1*	*Mata KlGDH1 KlGLT1 his3 ura3*	Valenzuela et al., 1995;
*KlWM37‐2*	*Mata Klgdh1*Δ*::natMx4 KlGLT1 his3 ura3*	This study
*KlWM37‐3*	*Mata KlGDH1 Klglt1*Δ::*kanMx4 his3 ura3*	Valenzuela et al., 1995
*KlWM37‐4*	*Mata Klgdh1*Δ*::natMx4, Klglt1*Δ::*kanMx4 his3 ura3*	This study
*KlWM37‐1‐1*	*KlWM37‐1/* YEpKD352 *his3*	This study
*KlWM37‐2‐1*	*KlWM37‐2/* YEpKD352 *his3*	This study
*KlWM37‐2‐2*	*KlWM37‐2/* YEpKD352*‐ScGDH1 his3*	This study
*KlWM37‐2‐3*	*KlWM37‐2/* YEpKD352*‐ScGDH3 his3*	This study
*KlWM37‐2‐4*	*KlWM37‐2/* YEpKD352*‐LkGDH1 his3*	This study
*KlWM37‐2‐5*	*KlWM37‐2/* YEpKD352*‐KlGDH1 his3*	This study

The *L. kluyveri Lkgdh1*Δ (*Lk156‐2*) mutant strain was obtained by replacing the ORF of *LkGDH1* with the selectable marker *kanMx4*. The *LkGDH1* gene was replaced by homologous recombination using a module containing the *kanMX4* cassette (1469 bp) flanked by 1067 bp of 5′UTR (−1074 to −7) and 1146 bp of 3′UTR (+1368 to +2514) sequences of *LkGDH1*. This module (3320 bp) was amplified by overlapped extension PCR with deoxyoligonucleotides 107 and 108 (−979 to +2341) using a template built up by three independent modules: (1) the *LkGDH1* 5′UTR amplified using the 101 and 102 deoxyoligonucleotides and genomic DNA from strain *Lk156‐1* as a template, (2) the *kanMX4* module which was amplified from the pFA6a plasmid using deoxyoligonucleotides 105 and 106, and (3) the *LkGDH1* 3′UTR amplified using deoxyoligonucleotides 103 and 104 and genomic DNA from strain *Lk156‐1* as a template. The PCR product was transformed into the *Lk156‐1* strain. Transformants were selected for G418 resistance (200 μg ml^−1^). Deoxyoligonucleotides 108‐1 and 108‐2 were used to verify the construction *Lkgdh1*Δ*::kanMx4,* these primers generated a module of 1517 bp (+216 of *kanMx4* to +2648 of 3′UTR of *LkGDH1*) using genomic DNA of the G418‐resistant transformants as a template. The deoxyoligonucleotides sequences are indicated in Table S1.

The *L. kluyveri Lkglt1*Δ (*Lk156‐3*) mutant strain was obtained by replacing the ORF of *LkGLT1* with the selectable marker *natMx4*. The *LkGLT1* gene was replaced by homologous recombination using a module containing the *natMX4* cassette (1477 bp) flanked by 1005 bp of 5′UTR (−1006 to −1) and 1006 bp of 3′UTR (+6438 to +7444) sequences of *LkGLT1*. This module (3488 bp) was amplified by overlapped extension PCR with deoxyoligonucleotides 111 and 114 using a template built up by three independent modules: (1) the *LkGLT1* 5′UTR amplified using 111 and 112 deoxyoligonucleotides, (2) the *natMx4* module flanked by homologous regions of the 5′UTR and 3′UTR of the *LkGLT1* gene, which was amplified from p4339 plasmid using deoxyoligonucleotides 109 and 110, and (3) the *LkGLT1* 3′UTR amplified using deoxyoligonucleotides 113 and 114. The PCR product was transformed into the *Lk156‐1* strain. Transformants were selected for nourseothricin resistance (100 μg ml^−1^). Deoxyoligonucleotides 115 and 116 were used to verify the construction *Lkglt1*Δ*::natMx4,* these deoxyoligonucleotides generated a module of 1012 bp (+875 of *natMx4* to +1887 of 3′UTR of *LkGLT1*) using genomic DNA of the nourseothricin‐resistant transformants as a template.

To generate the *L. kluyveri* (*Lk156‐4*) double‐mutant strain*,* the above described *Lkgdh1*Δ*::kanMx4* cassette was transformed into the *Lk156‐3* strain. Transformants were selected for G418 (geneticin) (200 μg ml^−1^) and nourseothricin resistance (100 μg ml^−1^). Deoxyoligonucleotides 108‐1 and 108‐2 were used to verify the construction as described above for the *Lk156‐2* mutant strain using genomic DNA of the G418 and nourseothricin‐resistant transformants as a template.

*K. lactis Klgdh1*Δ (*KlWM37‐2*) mutant strain was obtained by replacing the ORF of *KlGDH1* with the selectable marker *natMx4* **.** The *KlGDH1* gene was replaced by homologous recombination using a module containing the *natMx4* cassette (1578 bp) flanked by 1000 bp of 5′UTR (−1000 to −1) and 997 bp of 3′UTR (+1386 to +2383) sequences of *KlGDH1*. This module (3575 bp) was amplified by overlapped extension PCR with deoxyoligonucleotides 119 and 122 using a template built up by three independent modules: (1) the *KlGDH1* 5′UTR amplified using 119 and 120 deoxyoligonucleotides, (2) the *natMx4* module flanked by homologous regions of the 5′UTR and 3′UTR of the *KlGDH1* gene, which was amplified from p4339 plasmid using deoxyoligonucleotides 117 and 118, and (3) the *KlGDH1* 3′UTR amplified using deoxyoligonucleotides 121 and 122. The PCR product was transformed into the *KlWM37‐1* strain. Transformants were selected for nourseothricin resistance (100 μg ml^−1^). Deoxyoligonucleotides 123 and 124 were used to verify the construction *Klgdh1*Δ*::natMx4,* these deoxyoligonucleotides generated a module of 2030 bp (−277 of 5′UTR of *KlGDH1* to +1670 of 3′UTR of *KlGDH1*) using genomic DNA of the nourseothricin‐resistant transformants as a template.

To generate the *K. lactis Klgdh1*Δ *Klglt1*Δ double mutant strain (*KlWM37‐4*), the above described *Klgdh1*Δ*::natMx4* cassette was transformed into the *KlWM37‐3* strain (Valenzuela et al., 1995). Transformants were selected for G418 (geneticin) (200 μg ml^−1^) and nourseothricin resistance (100 μg ml^−1^). Deoxyoligonucleotides 123 and 124 were used to verify the construction as described above for the *KlWM37‐2* mutant strain using genomic DNA of G418 and nourseothricin‐resistant transformants as a template.

### 2.2 Growth conditions

Strains were routinely grown on minimal medium (MM) containing salts, trace elements, and vitamins following the formula of yeast nitrogen base (Difco). Sterilized glucose (2%, w/v) or ethanol (2%, w/v) was used as a carbon source. A quantity of 40 mmol/L ammonium sulfate or 5 mmol/L glutamate was used as a nitrogen source. Supplements needed to satisfy auxotrophic requirements were added at 0.1 mg ml^−1^. Cells were incubated at 30°C with shaking (250 rpm). Growth was monitored by measuring optical density at 600 nm (Thermo Fisher Scientific, Genesys 20 model 4001/4 spectrophotometer).

### 2.3 Construction of Plasmids Bearing the ScGDH1, ScGDH3, LkGDH1, or KlGDH1 Genes

All standard molecular biology techniques were followed as previously described (Sambrook, Fritsch, & Maniatis, 1989). The *ScGDH1, ScGDH3*,* LkGDH1*, and *KlGDH1* genes were PCR amplified together with their 5′ promoter sequence and cloned into either the pRS416 (*CEN6‐ARSH4‐URA3*), p*Lk‐*EE (*CEN6‐ARSH4‐LkURA3*) (provided by Dr. Lina Riego‐Ruiz), or YEpKD352 (pKD1 ori‐*KlURA3*), respectively (Colon et al., 2011; Wach, Brachat, Pohlmann, & Philippsen, 1994). Cloning into the pRS416 plasmid was made as follows: for the *ScGDH1* gene*,* a 2514 bp region between −850 bp upstream the start codon and +275 bp downstream the stop codon was amplified with deoxyoligonucleotides 133 and 134 using genomic DNA from the *S. cerevisiae* (*CLA1*) WT strain as a template; for the *ScGDH3* gene*,* a 2466 bp region between −780 bp upstream the start codon and +288 bp downstream the stop codon was amplified with deoxyoligonucleotides 135 and 136 using genomic DNA from the *S. cerevisiae* (*CLA1*) WT strain as a template; for the *LkGDH1* gene*,* a 2611 bp region between −920 bp upstream the start codon and +281 bp downstream the stop codon was amplified with deoxyoligonucleotides 137 and 138 using genomic DNA from the *L. kluyveri* (*Lk156‐1*) WT strain as a template; and for *KlGDH1,* a 2532 bp region between −874 bp upstream the start codon and +248 bp downstream the stop codon was amplified with deoxyoligonucleotides 139 and 140 using genomic DNA from the *K. lactis* (*KlWM37‐1*) WT strain as a template. The PCR products and pRS416 plasmid were digested with restriction enzymes (*Bam*HI/*Xho*I for *ScGDH1* and *ScGDH3, Bam*HI/*Sac*I for *LkGDH1* and *Bam*HI/*Xba*I for *KlGDH1*) and after gel purification were ligated.

Cloning into the p*Lk‐*EE plasmid was made following the same strategy: the *ScGDH1, ScGDH3*,* LkGDH1*, and *KlGDH1* genes products were (*Bam*HI/*Xho*I, *Bam*HI/*Sac*I, *Bam*HI/*Xba*I, and *Bam*HI/*Sma*I, respectively) digested and after gel purification were ligated. And for cloning into the YEpKD352 plasmid, the *ScGDH1, ScGDH3*,* LkGDH1* and *KlGDH1* genes products were (*Bam*HI/*Sac*I, *Bam*HI/*Sma*I, *Bam*HI/*Xba*I and *Bam*HI/*Xho*I, respectively) digested and after gel purification were ligated. The cloned genes were sequenced to check ORF integrity and after were transformed into the *CLA2*,* CLA5, Lk156‐2*, and *KlWM37‐2* strains as indicated in Table 1. Yeast strains (*S. cerevisiae, L. kluyveri*, and *K. lactis*) were transformed following a previously described method (Ito, Fukuda, Murata, & Kimura, 1983). Transformants were selected for uracil prototrophy on MM.

### 2.4 NADP‐GDH purification

#### 2.4.1 Cloning and expression

The *ScGDH1* and *ScGDH3* genes were PCR amplified using the deoxyoligonucleotides pairs 125/126 and 127/128, respectively, using genomic DNA of the *CLA1* WT strain as a template. PCR products and the pET‐28a(+) plasmid were *Nhe*I/*Xho*I digested and after gel purification were ligated. The *LkGDH1* gene was amplified with the deoxyoligonucleotides 129 and 130 using genomic DNA of the *Lk156‐1* WT strain as a template. PCR product and the pET‐28a(+) plasmid were *Nde*I/*Bam*HI digested and after gel purification were ligated. The *KlGDH1* gene was amplified with the deoxyoligonucleotides 131 and 132 using genomic DNA of the *KlWM37‐1* WT strain as a template. PCR product and the pET‐28a(+) plasmid were *Nhe*I/*Bam*HI digested and after gel purification ligated.

Ligations were transformed into the DH5α *E. coli* strain. After plasmid purification, correct cloning was verified by sequencing. For heterologous expression, the BL21 *E. coli* strain was transformed. Selected clones were grown in LB medium supplemented with 30 μg ml^−1^ of kanamycin incubated at 37°C with shaking (250 rpm). When the cultures reached an OD of 0.6 at 600 nm, the expression of the proteins was induced with 100 μmol/L of IPTG (Iso‐Propil‐Tio‐Galactoside), incubated 4 hr at 30°C with shaking (250 rpm), harvested by centrifugation at 1100*g* for 15 min, and the cellular pellet was stored at −70°C until used.

### 2.5 Whole cell soluble protein extract

Cells were thawed and resuspended in 20 ml of 30 mmol/L imidazol, 1 mmol/L EDTA, 1 mmol/L dithiothreitol, 1 mmol/L phenylmethylsulfonylfluoride (PMSF). Protein extracts were obtained by sonication (Ultrasonic Processor Model: VCX 130) with a tip sonicator keeping the tubes on ice; five cycles (60% amplitude, one second on and one second off for 1 min) with 1 min of incubation on ice between each cycle. After centrifugation at 1100*g* for 20 min at 4°C, the supernatant was stored at −20°C until used.

### 2.6 Affinity Chromatography

To purify the NADP‐GDH proteins, the supernatant was loaded on an equilibrated nickel column (Ni‐NTA Agarose 100, Thermo Fisher Scientific), which was then washed 10 times with 30 mmol/L imidazol. The protein was eluted with 500 mmol/L imidazol and stored at −20°C until used. Homogeneity of proteins was verified with a polyacrylamide gel electrophoresis 12% (SDS‐PAGE) stained with Coomassie Blue (Fig. S2).

### 2.7 Enzyme assay and protein determination

Whole yeast cell soluble protein extracts were prepared by sonication lysis of cell pellets harvested during exponential growth. The NADP‐GDH activity was assayed by the method of Doherty (Doherty, 1970)**.** Protein was measured by the method of Lowry (Lowry, Rosebrough, Farr, & Randall, 1951)**,** using bovine serum albumin as a standard.

### 2.8 Enzyme kinetics and analysis of kinetic data

NADP‐GDH activity was assayed for the reductive amination reaction at different concentrations of α‐KG (0.02–12 mmol/L), NADPH (5–500 μmol/L), or ammonium chloride (1–100 mmol/L) and at saturating concentrations of the remaining substrates (8 mmol/L α‐KG, 250 μmol/L NADPH, and 100 mmol/L ammonium chloride). The progress of the reaction was always kept below 5% conversion of the initial substrate. Measurements were made in 100 mmol/L Tris at pH 7.5 for *Sc*Gdh1, *Sc*Gdh3, and *Lk*Gdh1 or 0.1 mol/L potassium phosphate at pH 7.5 for *Kl*Gdh1. For experiments in which pH was 5.8, 25 mmol/L acetic acid, 25 mmol/L MES, and 50 mmol/L of TRIS or potassium phosphate at pH 5.8 was used as buffer. Kinetic data were analyzed by nonlinear regression using the program GraphPad Prism 5.00 (Software Inc.). All assays were performed at 340 nm, 30°C in a Varian Cary 50 spectrophotometer.

### 2.9 Glutamic inhibition

To study glutamic inhibition, were prepared protein extracts of *S. cerevisiae WT, Scgdh1Δ, L. kluyveri WT y K. lactis WT* strains grown on MM with ammonium sulfate as a nitrogen source and 2% glucose or ethanol as carbon source. Saturation curves were determined at the following glutamic concentrations: 0, 3, 5, 10, 15, 20, 50, 100, 200, 300, 400, 500, 600, 700, 800, 900, and 1000 mmol/L for every strain. At every glutamic concentration, the α‐KG, ammonium chloride and NADPH were fixed (8 μmol/L, 100 mmol/L, and 250 μmol/L, respectively). In order to select the inhibition model, the data were fitted to different models, with the program Dynafit. IC_50_ results were globally obtained with program GraphPad Prism 7.00 (Software Inc.).

### 2.10 Northern blot analysis

Northern blot analysis was carried out as previously described Struhl K. and Davis RW (1981). Total yeast RNA was prepared from 100 ml aliquots of cultures grown to an OD 600 nm of 0.6 in MM with ammonium sulfate as a nitrogen source and 2% glucose or ethanol as carbon source. PCR products were used as probes. For *ScGDH1*, a 645 bp product was amplified with deoxyoligonucleotides 141 and 142; for *ScGDH3,* a 1156 bp PCR product was amplified with deoxyoligonucleotides 143 and 144; 1200 bp fragment amplified using deoxyoligonucleotides 145 and 146 of *ScACT1* was used as internal loading standard; for *LkGDH1*, a 1180 bp product was amplified with deoxyoligonucleotides 147 and 148; 477 bp fragment amplified using deoxyoligonucleotides 149 and 150 of *Lk18s* was used as internal loading standard; for *KlGDH1*, a 1386 bp product was amplified with deoxyoligonucleotides 151 and 152 and 477 bp fragment amplified using deoxyoligonucleotides 153 and 154 of *Kl18s* was used as internal loading standard. Blots were scanned with ImageQuant 5.2 program (Molecular Dynamics).

### 2.11 Nucleosome scanning assay (NuSA)

The nucleosome scanning assay was made to see the chromatin organization *ScGDH1, ScGDH3*,* LkGDH1* y *KlGDH1* promoter, and the procedure to the study of the positioning of nucleosomes on promoters was made as described by Infante et al. 2011. When the cultures reached an OD of 0.6 at 600 nm, genetic DNA was obtained of *Cla1, Cla2*,* KlWM37‐1*, and *Lk156‐1* strains grown on minimal medium with ammonium sulfate as nitrogen source and 2% glucose or ethanol as carbon source. Cells were treated with formaldehyde (1% final concentration) for 20 min at 37°C and then glycine (125 mmol/L final concentration) for 5 min at 37°C. Formaldehyde‐treated cells were harvested by centrifugation, washed with Tris‐buffered saline, and then incubated in Buffer Z2 (1 mol/L Sorbitol, 50 mmol/L Tris‐Cl at pH 7.4, 10 mmol/L β‐mercaptoethanol) containing 2.5 mg of zymolase 20T for 20 min at 30°C on rocker platform. Spheroplast were pelleted by centrifugation at 3000*g*, and resuspended in 1.5 ml of NPS buffer (0.5 mmol/L Spermidine, 0.075% NP‐40, 50 mmol/L NaCl, 10 mmol/L Tris pH 7.4, 5 mmol/L MgCl_2_, 1 mmol/L CaCl_2_, 1 mmol/L β‐mercaptoethanol). Samples were divided into three 500 μl aliquots that were then digested with 22.5 U of MNase (Nuclease S7 from Roche) at 50 min at 37°C. Digestions were stopped with 12 μl of Stop buffer (50 mmol/L EDTA and 1% SDS) and were treated with 100 μg of proteinase K at 65°C over night. DNA was extracted twice by phenol/chloroform and precipitated with 20 μl of 5 mol/L NaCl and equal volume of isopropanol for 30 min at −20°C. Precipitates were resuspended in 40 μl of TE and incubated with 20 μg RNase A for 1 hr at 37°C. DNA digestions were separated by gel electrophoresis from a 1.5% agarose gel. Monosomal bands (150 bp) were cut and purified by Wizard SV Gel Clean‐Up System Kit (Promega, REF A9282). DNA samples were diluted 1:30 and used in quantitative polymerase chain reactions (qPCR) to quantify the relative MNase protection of each *ScGDH1, ScGDH3*,* LkGDH1* y *KlGDH1* template. qPCR analysis was performed using a Corbett Life Science Rotor Gene 6000 machine. The detection dye used was SYBR Green (2× KAPA SYBR FAST qBioline and Platinum SYBR Green from Invitrogen). Real‐time PCR was carried out as follows: 94° for 5 min (1 cycle), 94° for 15 s, 58° for 20 s, and 72° for 20 s (35 cycles). Relative protection was calculated as a ratio to the control *ScVCX1, LkVCX1*, and *KlVCX1* template found within a well‐positioned nucleosome in +250 bp of the ORFs. The PCR primers amplify from around −950 to +250 bp (Table S3) of *ScGDH1, ScGDH3*,* LkGDH1* y *KlGDH1* locus whose coordinates are given relative to the ATG (+1).

### 2.12 Metabolite extraction and analysis

Cell extracts were prepared from exponentially growing cultures. Samples used for intracellular amino acid determination were treated as previously described (Quezada et al., 2008).

### 2.13 Phylogenetic analysis

A total of 26 taxa were used in the analysis, including two ascomycetes as outgroup (Table S2). Glutamate dehydrogenase sequences were obtained from YGOB (http://ygob.ucd.ie) (Byrne & Wolfe, 2005) and ESEMBLFungi (http://fungi.ensembl.org/index.html) (Kersey et al., 2016) databases using *Sc*Gdh1 sequence as query.

The bootstrap neighbor‐joining tree (500 replicates) was constructed with *MEGA* version 6 software (http://www.megasoftware.net/) (Tamura, Stecher, Peterson, Filipski, & Kumar, 2013), based on the sequence alignment constructed with the multi‐alignment program Muscle. Alternatively, phylogenetic analysis was also conducted with Maximum Likelihood method, in order to improve the accuracy of the phylogenetic analysis.

## 3 Results

### 3.1 NADP‐GDH is the main glutamate‐producing pathway in *S. cerevisiae, L. kluyveri*, and *K. lactis*

To analyze the relative contribution of glutamate dehydrogenases (NADP‐GDH) and glutamate synthase (GOGAT) to glutamate biosynthesis, mutant strains were constructed in which the genes encoding for the NADP‐GDH (*GDH1/GDH3*) or GOGAT (*GLT1*) were inactivated (Table 1). Growth rates of these mutants were determined on minimal media with glucose or ethanol as carbon sources and ammonium as nitrogen source (Table 2). In the three yeast species, inactivation of the NADP‐GDH‐encoding genes resulted in a strong reduction in growth rate on both carbon sources (from 60% to 80% relative to the corresponding WT strains) indicating that the proteins *Sc*Gdh1/*Sc*Gdh3 in *S. cerevisiae*,* Lk*Gdh1 in *L. kluyveri* and *Kl*Gdh1 in *K. lactis*, are the main contributors to glutamate production under the conditions studied. The glutamine synthetase‐GOGAT pathway in *S. cerevisiae* made a marginal contribution to glutamate production under the conditions studied because the *glt1Δ* mutant strain grew as well as the wild‐type strain (Table 2). However, in *L. klyveri* and *K. lactis*, the GOGAT pathway made a significant contribution since inactivation of the *GLT1* genes, resulted in reduction of growth rates ranging from 25% to 60% (Table 2). As expected, the mutants lacking NADP‐GDH and GOGAT‐encoding genes were full glutamate auxotrophs (Table 2). In the pre‐WGD species, only one gene is responsible for the NADP‐GDH activity because inactivation of either *LkGDH1* or *KlGDH1* resulted in complete lack of this activity (Table 2). In agreement with previous reports (DeLuna et al., 2001), the contribution of *Sc*Gdh3 was evident on ethanol but not on glucose.

**Table 2:** Growth rates and NADP‐GDH specific activities

Strains	Growth rates				Specific activities			
	Glucose	Glucose +Glu	Ethanol	Ethanol +Glu	Glucose	Glucose +Glu	Ethanol	Ethanol +Glu
*S. cerevisiae*								
WT *ScGDH1 ScGDH3 ScGLT1*	100	100	100	100	0.741 (0.05)	0.710 (0.05)	0.818 (0.05)	0.801 (0.06)
*Scgdh1*Δ *ScGDH3 ScGLT1*	37	98	60	95	0.048 (0.02)	0.046 (0.07)	0.459 (0.04)	0.435 (0.04)
*ScGDH1 Scgdh3*Δ *ScGLT1*	91	93	80	91	0.749 (0.06)	0.698 (0.03)	0.938 (0.03)	0.704 (0.04)
*Scgdh1*Δ *Scgdh3*Δ *ScGLT1*	20	96	35	95	ND	ND	ND	ND
*ScGDH1 ScGDH3 Scglt1*Δ	97	94	94	92	0.654 (0.03)	0.694 (0.08)	0.901 (0.06)	0.781 (0.02)
*Scgdh1*Δ *Scgdh3*Δ *Scglt1*Δ	ND	74	ND	78	ND	ND	ND	ND
*L. kluyveri*								
WT *LkGDH1 LkGLT1*	100	100	100	100	0.252 (0.02)	0.279 (0.07)	0.465 (0.03)	0.424 (0.02)
*Lkgdh1*Δ *LkGLT1*	17	96	20	95	ND	ND	ND	ND
*LkGDH1 Lkglt1*Δ	70	81	59	77	0.229 (0.03)	0.253 (0.03)	0.418 (0.05)	0.451 (0.08)
*Lkgdh1*Δ *Lkglt1*Δ	ND	72	ND	73	ND	ND	ND	ND
*K. lactis*								
WT *KlGDH1 KlGLT1*	100	100	100	100	0.431 (0.03)	0.489 (0.08)	0.645 (0.04)	0.682 (0.05)
*Klgdh1*Δ *KlGLT1*	43	98	32	97	ND	ND	ND	ND
*KlGDH1 Klglt1*Δ	75	76	64	62	0.446 (0.04)	0.434 (0.04)	0.593 (0.02)	0.587 (0.08)
*Klgdh1*Δ *Klglt1*Δ	ND	78	ND	65	ND	ND	ND	ND

Glu, glutamate. ND, not detected. Numbers in parentheses are standard deviations.

Growth rates values are shown relative to the WT strains: for *S. cerevisiae* 0.258 hr^−1^ and 0.156 hr^−1^; for *L. kluyveri*, 0.179 hr^−1^ and 0.073 hr^−1^; for *K. lactis,* 0.322 hr^−1^ and 0.264 hr^−1^ on glucose and ethanol, respectively, in the absence of glutamate. However, in the presence of this amino acid, growth rates of the WT strains were for *S. cerevisiae* 0.261 hr^−1^ and 0.154 hr^−1^; for *L. kluyveri*, 0.182 hr^−1^ and 0.075 hr^−1^; for *K. lactis*, 0.325 hr^−1^ and 0.268 hr^−1^ on glucose and ethanol, respectively. For the growth rates, the standard deviations of at least three independent cultures were less than 5%. Specific activity values are shown in μmol min^−1^ mg^−1^.

When glutamate was supplemented to the growth media, *gdh*Δ mutant strains recovered wild‐type growth (Table 2). However, this was not the case for the *L. kluyveri* and *K. lactis glt1*Δ mutant strains, which did not recover wild‐type growth rate by glutamate addition. As previously reported, in addition to glutamate biosynthesis, GOGAT plays other role, which has been found to be critical for the maintenance of the redox balance and cytosolic NADH homeostasis (Guillamon et al., 2001).

### 3.2 Glutamate is not a negative regulator of the *S. cerevisiae*,* L. kluyveri and K. lactis,* NADP‐GDHs

To further analyze the regulation of the NADP‐GDH enzymes, specific activities in the presence of glutamate were determined. The use of 5 mmol/L glutamate as nitrogen source did not result on a reduction in NADP‐GDH specific activities as compared to those observed when ammonium sulfate was used (Table 2). Furthermore, when clarified extracts from the WT strains were analyzed in the presence of increasing glutamate concentrations, the half inhibitory concentrations were in the range of 376–681 mmol/L (Fig. S1); this range is much higher than the estimated cytosolic glutamate concentration, 9–46 mmol/L (Table 4). These results indicate that glutamate does not trigger strong negative regulatory mechanisms (e.g., repression of transcription or feedback inhibition) of glutamate biosynthesis under the conditions studied.

### 3.3 *S. cerevisiae, L. kluyveri*, and *K. lactis* showed different patterns of carbon source‐dependent transcriptional regulation of NADP‐GDH encoding genes

**Figure 1:**
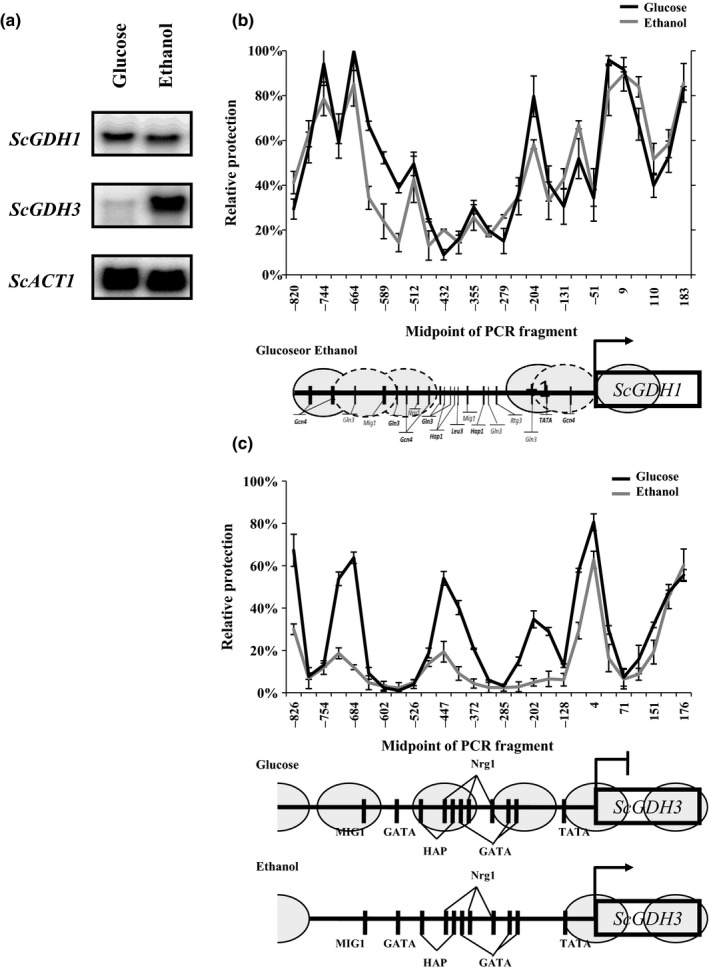
*ScGDH1* and *ScGDH3* NuSA and *e*xpression profile. (a) Northern blot analysis shown expression profile of *ScGDH1* gene*, ScGDH3* gene, and *ScACT1* gene as control in glucose or ethanol as carbon source. (b) Nucleosome Scanning Assay (NuSA) *ScGDH1* gene promoter in glucose (black line) or ethanol (gray line); and (c) Nucleosome Scanning Assay (NuSA) *ScGDH3* gene promoter in glucose (black line) or ethanol (gray line); nucleosomes are shown in gray ovals and black vertical lines shown DNA‐binding sites. Error bars represent the standard deviations

**Figure 2:**
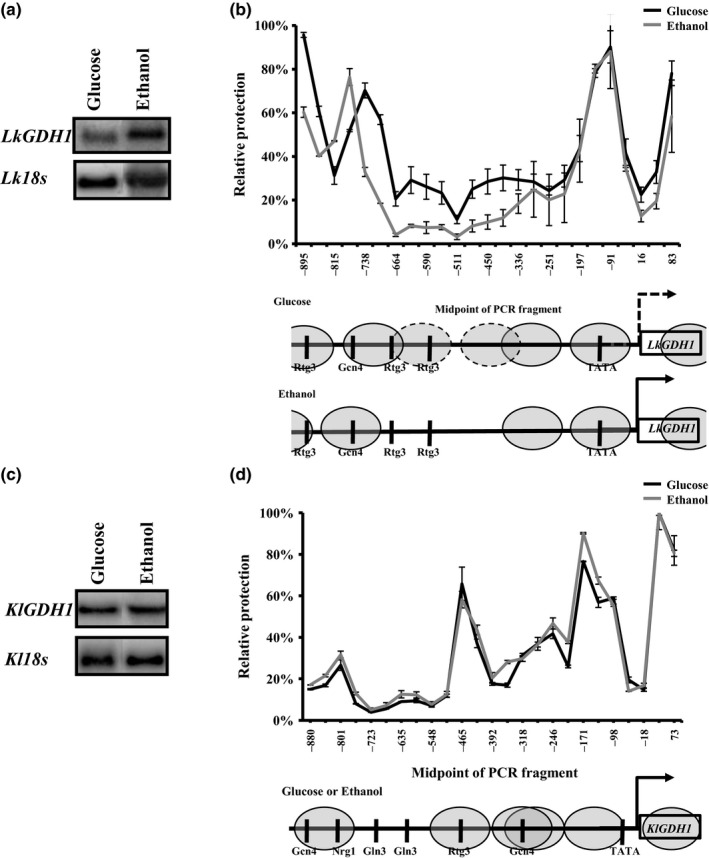
*LkGDH1* and *KlGDH1* NuSA and *e*xpression profile. (a) Northern blot analysis shown expression profile of *LkGDH1* gene and (c) Northern blot analysis shown expression profile *KlGDH3* gene*,* in both cases *Lk*18s or *Kl*18s ribosomal gene was used as control in glucose or ethanol as carbon source. (b) Nucleosome scanning assay (NuSA) *LkGDH1* gene promoter in glucose (black line) or ethanol (gray line); and (d) Nucleosome Scanning Assay (NuSA) *KlGDH1* gene promoter in glucose (black line) or ethanol (gray line); nucleosomes are shown in gray ovals and black vertical lines shown DNA binding sites. Error bars represent the standard deviations

In order to deepen the studies with regard to carbon source‐dependent regulation of the NADP‐GDH enzymes, specific activities, transcript levels of the corresponding genes and nucleosome positioning on the promoter regions were analyzed. As previously reported, transcription of the *ScGDH3* gene was higher on ethanol as carbon source compared to that observed on glucose (Figure 1a) (Avendano et al., 2005 and Riego et al., 2002). This was accompanied by nucleosome clearance on the −754 bp to −128 bp *ScGDH3* promoter region (Figure 1c) (Avendano et al., 2005). Transcript levels of the *ScGDH1* gene and nucleosome positioning on the promoter region were similar on both carbon sources (Figure 1a and b). Albeit in *S. cerevisiae*, the overall activity was similar on both carbon sources (Table 2), and the relative contribution of the *Sc*Gdh3 isoform was higher on ethanol than on glucose. When the mutant strain *Scgdh1*Δ was grown on ethanol, the *Sc*Gdh3‐specific activity was 10‐fold increased (Table 2). This is in accordance with previous results (DeLuna et al., 2001) demonstrating the observed differential contributions of each enzyme to growth rates (Table 2). When *L. kluyveri* and *K. lactis* were grown on ethanol as carbon source, activities were increased 80% and 50%, respectively, compared to those observed on glucose (Table 2). In *L. kluyveri*, transcription of the *LkGDH1* gene was slightly increased on ethanol as carbon source concomitantly with nucleosome clearance on the −738 bp to −336 bp promoter region (Figure 2a and b), whereas that transcriptional levels and nucleosome positioning of *KlGDH1* gene no change in carbon sources studied (Figure 2c and d).

### 3.4 *ScGDH1/LkGDH1* and *ScGDH3* */* *KlGDH1* gene pairs showed distinctive heterologous complementation patterns

**Figure 3:**
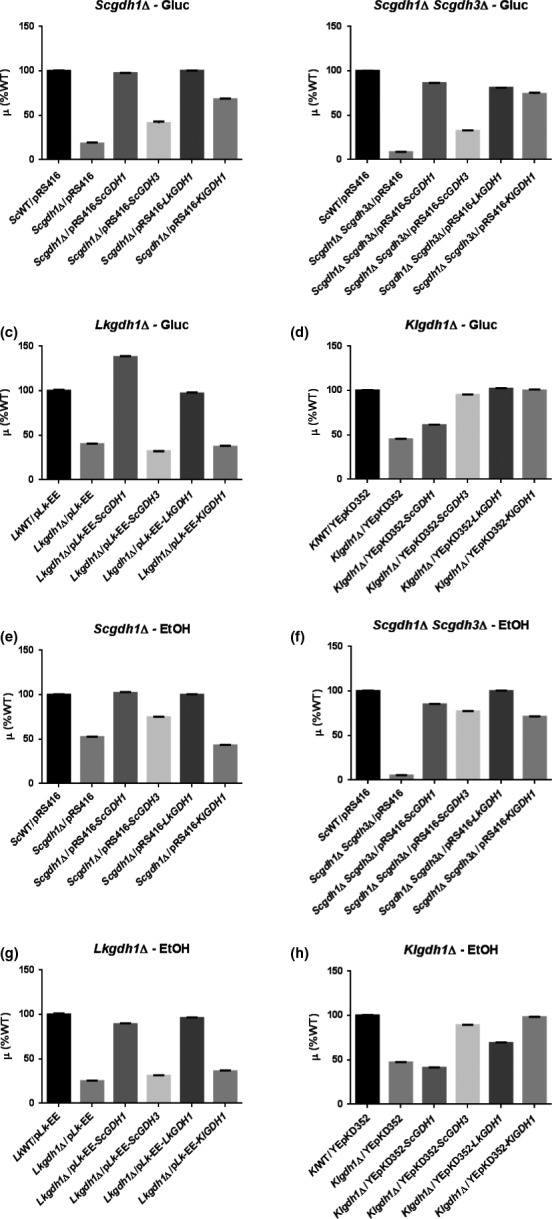
Complementation tests. Growth rates values are shown relative to the WT strains carrying the empty plasmid: for *S. cerevisiae* 0.23 hr^−1^ and 0.15 hr^−1^; for *L. kluyveri*, 0.12 hr^−1^ and 0.07 hr^−1^; for *K. lactis*, 0.35 hr^−1^ and 0.14 hr^−1^ on glucose and ethanol, respectively. In all cases, standard deviations of at least three independent cultures were less than three percent. For ectopic expression, the plasmid pRS416 was used for *S. cerevisiae*, the p*L* *k*‐EE for *L. kluyveri* and the YEpKD352 for *K. lactis* as described in Experimental procedures

To determine to what extent the various NADP‐GDHs were specialized to the metabolic peculiarities of the species they belong to, heterologous complementation tests were made. To this end, *ScGDH1, ScGDH3, LkGDH1*, and *KlGDH1* were cloned in the low copy‐number plasmids pRS416, p*Lk*‐EE, and YEpKD352 for expression in *S. cerevisiae*,* L. kluyveri* or *K. lactis*, respectively, under the transcriptional control of their native promoters as described in Experimental procedures. Mutant strains lacking NADP‐GDH activity were selectively transformed with the pertinent plasmids and their growth rates were compared (Figure 3).

As expected, homologous expression of *ScGDH1* in the *S. cerevisiae Scgdh1*Δ mutant strain (third bar in Figure 3a and e), of *LkGDH1* in the *L. kluyveri Lkgdh1*Δ mutant strain (fifth bar in Figure 3c and g) and of *KlGDH1* in the *K. lactis Klgdh1*Δ mutant strain (last bar in Figure 3d and h), restored wild‐type growth when strains were grown on either glucose or ethanol. Homologous expression of the *S. cerevisiae ScGDH3* gene resulted in a discrete but significant complementation of the *gdh1*Δ mutant strain on glucose (fourth bar in Figure 3a and b) and in almost full complementation on ethanol as carbon source (fourth bar in Figure 3e and f). These last results are in agreement with the proposed specialized role of *Sc*Gdh3 during respiratory conditions and with the carbon source‐dependent regulation of *ScGDH3* gene (Avendano et al., 2005; DeLuna et al., 2001).

Heterologous expression of the *ScGDH1* and *LkGDH1*genes resulted in similar patterns of complementation in *S. cerevisiae* and *L. kluyveri* where expression of these genes restored growth rates close to those of the WT reference strains (third bar in Figure 3c and g, and fifth bar in a, b, e and f). This effect, however, was not observed upon expression in *K. lactis* (Figure 3d and h). In this yeast, expression of the *L. kluyveri LkGDH1* gene resulted in full complementation of the *Klgdh1*Δ mutant strain on glucose and in partial complementation on ethanol (fifth bars in Figure 3d vs. h), whereas expression of the *S. cerevisiae ScGDH1* gene resulted in poor growth on either glucose or ethanol (third bars in Figure 3d and h). Interestingly, expression of the *ScGDH1* and *LkGDH1* genes also showed a similar trend when expressed on *K. lactis* grown on ethanol. In this case, however, the corresponding growth rates were significantly lower than those of the WT strain (third and fifth bars in Figure 3h), which showed the opposite trend to that observed upon expression on *S. cerevisiae* or *L. kluyveri*.

Ectopic expression of the *ScGDH3* and *KlGDH1* genes also showed a similar trend in the complementation experiments. When expressed on *L. kluyveri*, they did not improve growth of the *Lkgdh1*Δ mutant strain (fourth and sixth bars in Figure 3c and g). When expressed on *S. cerevisiae*, they showed significant complementation but the growth rates were still lower than those of the corresponding WT strains (fourth and sixth bars in Figure 3a, b, e and f). Transcriptional repression of the *ScGDH3* gene on glucose (Avendano et al., 2005), may have contributed to the low levels of complementation of the *ScGDH3* gene in the *S. cerevisiae* mutant strain grown on glucose (fourth and sixth bars in Figure 3a and b). Furthermore, when the *ScGDH3* gene was expressed in *K. lactis*, it fully complemented growth of the *Klgdh1*Δ mutant strain at levels similar to those of the endogenous *KlGDH1* gene (fourth and sixth bars in Figure 3d and h).

**Figure 4:**
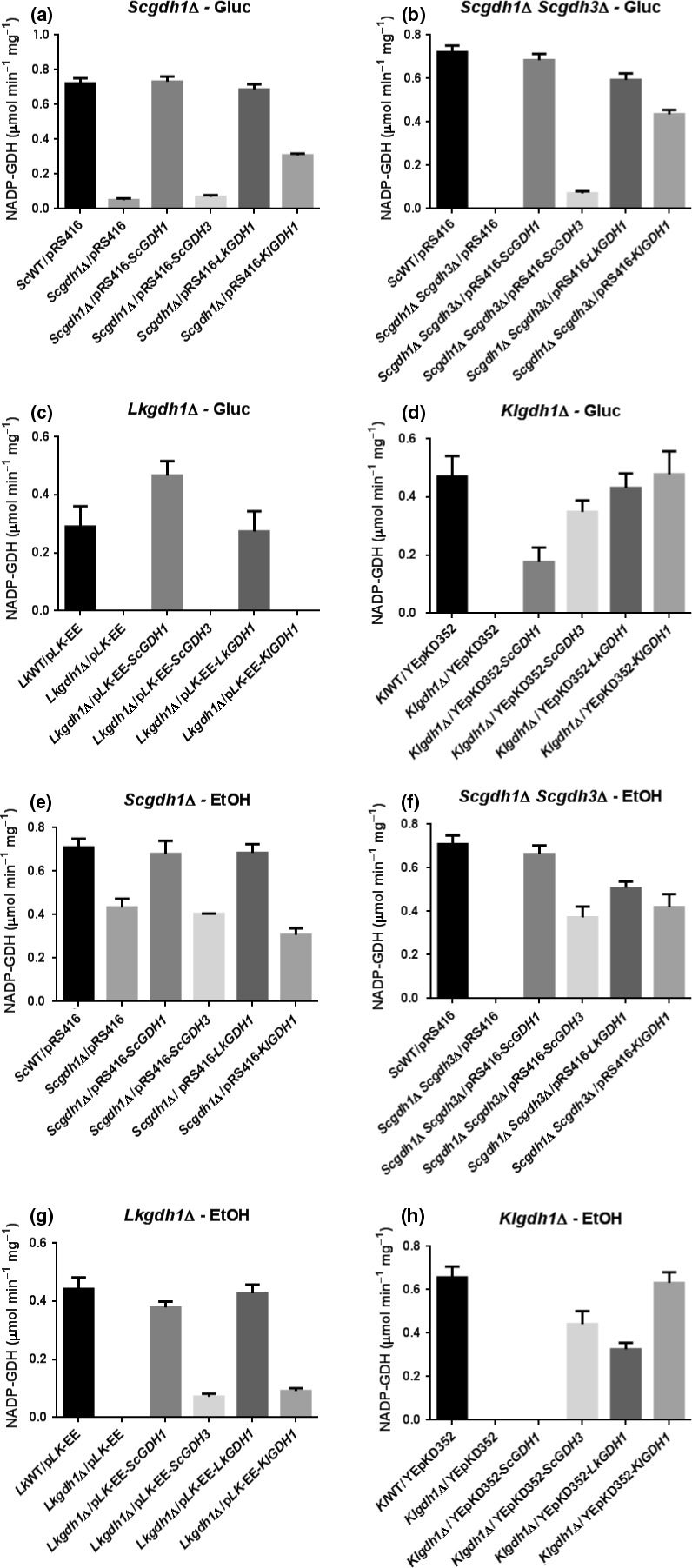
NADP‐GDH specific activities in complemented strains. NADP‐GDH specific activities values are shown in μmol min^−1^ mg^−1^. ND, not detected. Numbers in parentheses are standard deviations of at least three independent cultures

These results suggest that peculiar transcriptional and/or kinetic regulatory mechanisms cluster the *ScGDH1* and *LkGDH1* genes, or their encoded proteins, in a separate group from the *ScGDH3* and *KlGDH1* genes. To determine to what extent the amount of active enzyme is responsible of this effect, the NADP‐GDH specific activities were determined (Figure 4). As expected, the non‐complemented *Scgdh1*Δ *Scgdh3*Δ double‐mutant strain, as well as the *Lkgdh1*Δ and *Klgdh1*Δ single mutants did not show detectable activity (lack of a second bar in Figure 4b, c, d, f, g and h). Growth of these mutant strains, which are devoid of NADP‐GDH activity, may have involved the GOGAT‐dependent glutamate‐producing pathway (Magasanik, 2003). In the *Scgdh1*Δ single mutant strain, however, presence of the *ScGDH3* gene was responsible of a very low activity on glucose and a significant activity on ethanol (second bars in Figure 4a and e), this was consistent with the induction of the *ScGDH3* gene under respiratory conditions (Figure 1a). In general, a clear correlation between growth rate and NADP‐GDH activity was observed (Figure 3 vs. 4) indicating that regulatory mechanisms determining synthesis of NADP‐GDH (e.g., transcriptional and translational) are similar between *KlGDH1* and *ScGDH3* and between *LkGDH1* and *ScGDH1*. However, in the *K. lactis‐*mutant strain grown on glucose, expression of the *LkGDH1* gene restored growth rate and the specific activity to levels similar to those observed when the homologous *KlGDH1*gene was expressed (fifth and sixth bars in Figures 3d and 4d) and a significant complementation was also observed on ethanol (fifth and sixth bars in Figures 3h and 4h) suggesting that, in *K. lactis*, expression of the *LkGDH1* gene is similar to that of the homologous *KlGDH1*gene. Reciprocal expression, however, resulted in poor growth and in absent or very low specific activity (sixth bars in Figures 3c, g and 4c, g), indicating that the contribution to the heterologous complementation capacity of *LkGDH1* and *KlGDH1* genes is mainly determined by the levels of active enzyme and not by their kinetic properties. Additionally, the lack of reciprocal complementation between these genes indicates that mechanisms determining synthesis of the NADP‐GDHs are regulated differently in *K. lactis* and *L. kluyvery* in spite of the close phylogenetic relationship between these species Figure 6).

Unexpectedly, NADP‐GDH activity was not detected in three complemented strains: the *Lkgdh1*Δ mutant strain grown on glucose bearing the *ScGDH3* and *KlGDH1* genes (lack of fourth and sixth bars in Figure 4C), and the *Klgdh1*Δ mutant strain grown on ethanol bearing the *ScGDH1* gene (lack of a third bar in Figure 4h). This suggested that expression of the heterologous genes in these strains was very low and below the detection limit. Interestingly, heterologous complementation of *K. lactis* with the *S. cerevisiae ScGDH1* and *ScGDH3* genes resulted in higher activities of *Sc*Gdh3 than those observed for *Sc*Gdh1 (third and fourth bars in Figure 4d and h) and this effect was similar to the relative contribution of the two isoforms observed in the WT strain grown on ethanol (Table 2).

### 3.5 *K. lactis KlGdh1* and *S. cerevisiae Sc*Gdh3 isoforms showed cooperativity for α‐KG utilization, whereas *L. kluyveri LkG*dh1 and *S. cerevisiae Sc*Gdh1 isoforms showed hyperbolic kinetics

In order to analyze the NADP‐GDHs biochemical characteristics and find additional elements that could contribute to better understand the results obtained in the complementation tests, substrate utilization kinetics was studied. The His‐tagged *Sc*Gdh1, *Sc*Gdh3, *Lk*Gdh1, and *Kl*Gdh1 enzymes were purified to electrophoretic homogeneity after heterologous expression in *E. coli* (Fig. S2). Apparent molecular masses of the *Sc*Gdh1, *Sc*Gdh3, *Lk*Gdh1, and *Kl*Gdh1 monomers, respectively, were: 53 kD, 55 kD, 54 kD, and 54 kD. In all cases, values are close to those expected. Initial velocity measurements were made at different substrate concentrations (α‐KG, NADPH and ammonium chloride); when the amount of one substrate was varied, the other two were kept at saturating concentrations. The reaction was assayed as previously reported (DeLuna et al., 2001).

**Figure 5:**
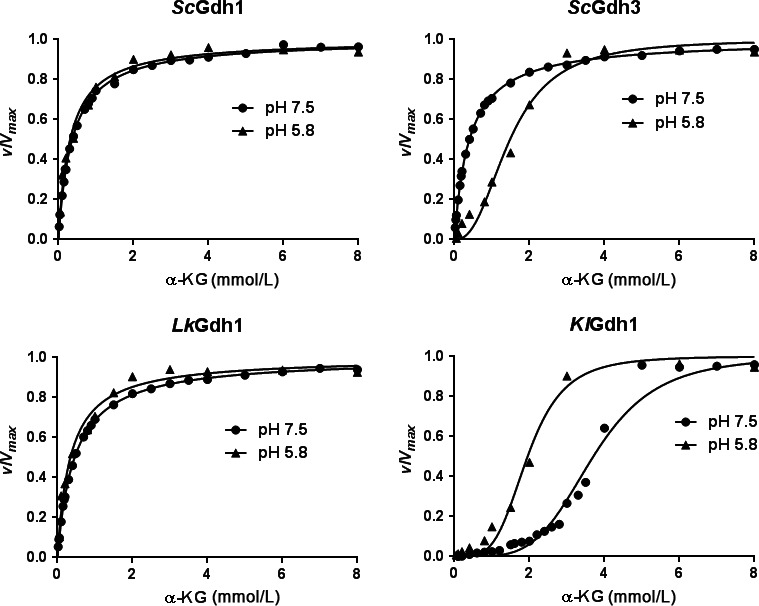
Effect of pH on the NADP‐GDH kinetic responses to α‐KG saturation. Initial velocities are shown as fractions of the corresponding *V*_*max*_ at different α‐KG concentrations. The reductive amination reaction was measured in pure recombinant proteins from *S. cerevisiae* (*Sc*Gdh1 and *Sc*Gdh3), *L. kluyveri* (*Lk*Gdh1), and *K. lactis* (*Kl*Gdh1). The corresponding kinetic parameters are shown in Table 3

The responses to increasing substrate concentrations were heterogeneous: they varied from hyperbolic for the three substrates in the *S. cerevisiae* isoform *Sc*Gdh1 and the *L. kluyveri* enzyme *Lk*Gdh1, to sigmoidal for the three substrates in the *K. lactis* enzyme *Kl*Gdh1 (Figure 5, S3 and S4). The *S. cerevisiae* isoform *Sc*Gdh3, however, showed hyperbolic response for the three substrates at pH 7.5, but sigmoidal for α‐KG at pH 5.8, in agreement with a previous study in which the nontagged homologous purified protein was used (DeLuna et al., 2001). This pH‐dependent difference in the shape of the α‐KG saturation curve was only observed for the *Sc*Gdh3 isoform (Figure 5, S3 and S4). Experimental data from the hyperbolic or sigmoidal responses were fitted to the Michaelis–Menten or the Hill equations, respectively, and the resulting kinetic parameters are shown in Table 3. At pH 7.5, the four enzymes showed similar turnover numbers, which indicate the catalytic events per unit of time (*k*_cat_), and similar affinities for NADPH and ammonium (Table 3). The *Sc*Gdh1, *Sc*Gdh3, and *Lk*Gdh1 isoforms also showed similar affinities for α‐KG, showing similar *K*_m‐αKG_ values. However, *Kl*Gdh1 showed lower affinity and strong cooperativity for α‐KG utilization, its affinity constant (*S*_0.5_) for α‐KG was about eight times higher than the *K*_m‐αKG_ of the other enzymes (Table 3) and the fitted Hill number (*n*_H‐α‐KG_) resulted to be 4.4. These *Kl*Gdh1 characteristics were also observed at pH 5.8 and, at this pH value, were also shared by the *Sc*Gdh3 isoform. At the acidic pH, the *k*_cat_ value of the *K. lactis* enzyme resulted to be three times lower than that observed at pH 7.5 (Table 3). Enzymatic activities of the *Lk*Gdh1 and *Kl*Gdh1 enzymes were assayed at different pH values ranging from 5 to 9. Maximum activities were observed at pH 7.0 (data not shown), which is close to the 6.8 value reported for the *S. cerevisiae* isoforms (DeLuna et al., 2001).

**Table 3:** Kinetic parameters of the studied NADP‐GDHs

Enzyme	pH 7.5							pH 5.8		
	*k*_cat_ (s^−1^)	*K*_*m*‐α‐KG_ (mmol/L)	*n*_*H‐* α‐KG_	*K*_*m‐NADPH*_ (μmol/L)	*n*_*H‐NADPH*_	*K*_*m‐NH4+*_ (mmol/L)	*n*_*H‐NH4+*_	*k*_cat_ (s^−1^)	*K*_*m*‐ α‐KG_ (mmol/L)	*n*_*H‐* α‐KG_
*Sc*Gdh1	13	0.37		45		8.6		12	0.31	
*Sc*Gdh3	14	0.4		42		6.9		13	*S*_0.5_=1.48	2.4
*Lk*Gdh1	20	0.46		46		7.4		10	0.34	
*Kl*Gdh1	21	*S*_0.5_=3.61	4.4	*S*_0.5_=39	1.8	*S*_0.5_=21.4	2.7	6	*S*_0.5_=1.95	4

Data were fitted to the Michaelis–Menten equation except for the *Sc*Gdh3 isoform at pH 5.8 and the *Kl*Gdh1 enzyme which were fitted to the Hill equation. Standard errors for the fitted parameters at pH 7.5 were lower than 8% and, at pH 5.8, lower than 16%. For all the fitting analyses, the *R* ^2^ value was higher than 0.982.

In order to get some insight into the in vivo kinetic regulation of the NADP‐GDHs, the α‐KG and glutamate intracellular pools were determined (Table 4). Similar α‐KG values were detected in the three yeast species, except for *L. kluyveri* grown on ethanol which showed much lower α‐KG content, which was probably associated with the very low growth rate observed for this yeast (Table 2). The estimated α‐KG cytosolic concentrations in *S. cerevisiae* and *L. kluyveri* were in the range of 0.1–1.14 mmol/L which is close to 0.4–1.76 mmol/L, the reported values for *S. cerevisiae* (Cueto‐Rojas, Maleki Seifar, Ten Pierick, Heijnen, & Wahl, 2016; Hans, Heinzle, & Wittmann, 2003). To estimate these concentrations, a cell volume of 29 μm^3^ of which 75% of corresponded to cytosol was used (Kitamoto, Yoshizawa, Ohsumi, & Anraku, 1988), and to compare them with the reported values, a cell volume of 1.7 ml/g cell dry weight was used (Zhang et al., 2015). The estimated α‐KG physiological concentrations correspond to 0.3–3 *K*_m‐α‐KG_ (Tables 3 and 4). At these concentrations, the catalytic rates of the *Sc*Gdh1, *Sc*Gdh3, and *Lk*Gdh1 enzymes were highly responsive to changes in substrate concentration (Figure 3). In *K. lactis*, the α‐KG estimated concentration was 0.7 mmol/L, which is around one‐fourth of the *S*_0.5‐α‐KG_ (Tables 3 and 4). At this concentration, and because of its strong cooperativity, the *Kl*Gdh1 enzyme catalytic rate was not highly responsive to changes in substrate concentration (Figure 3).

**Table 4:** Intracellular metabolite pools

	α‐KG				Glutamate			
	Glucose		Ethanol		Glucose		Ethanol	
	(nmol×10^8^ cells)	(mmol/L)	(nmol×10^8^ cells)	(mmol/L)	(nmol×10^8^ cells)	(mmol/L)	(nmol×10^8^ cells)	(mmol/L)
*S. cerevisiae*	1.0 (0.05)	0.5	2.4 (0.21)	1.1	100 (4)	46	81 (14)	37
*L. kluyveri*	2.1 (0.12)	1.0	0.2 (0.03)	0.1	20 (1)	9	98 (8)	45
*K. lactis*	1.5 (0.23)	0.7	1.4 (0.13)	0.7	25 (1)	11	40 (5)	18

Concentrations in mmol/L represent the cytosolic pool and were estimated considering a cell volume of 29 μm^3^ of which 75% of corresponded to cytosol (Kitamoto et al., 1988). Numbers in parentheses are standard deviations.

The intracellular glutamate content observed on ethanol as carbon source was similar to that observed on glucose in *S. cerevisiae* and *K. lactis*, although the pools in the former were higher than in the latter (Table 4). In *L. kluyveri*, however, the intracellular glutamate pool was much higher on ethanol than on glucose. This could be the results of the high‐affinity *Lk*Gdh1 has for α‐KG, driving intermediate flux to glutamate biosynthesis, which could result in decreased growth rate due to a restriction of carbon flow through Krebs Cycle and limited energy production resulting in low α‐KG intracellular concentration (Table 4). The estimated glutamate intracellular concentrations was found in the range of 9–46 mmol/L (Table 4), which are near to the 30–80 mmol/L, reported values for *S. cerevisiae* (Cueto‐Rojas et al., 2016; Hans et al., 2003). At these concentrations, glutamate does not exert a significant inhibitory effect over the NADP‐GDH activity as indicated by the high IC_50_ values, which were detected (Fig. S1).

### 3.6 Phylogenetic analysis of NADP‐GDH‐sequences revealed that the evolutionary origin of these proteins does not correlate with their kinetic properties

**Figure 6:**
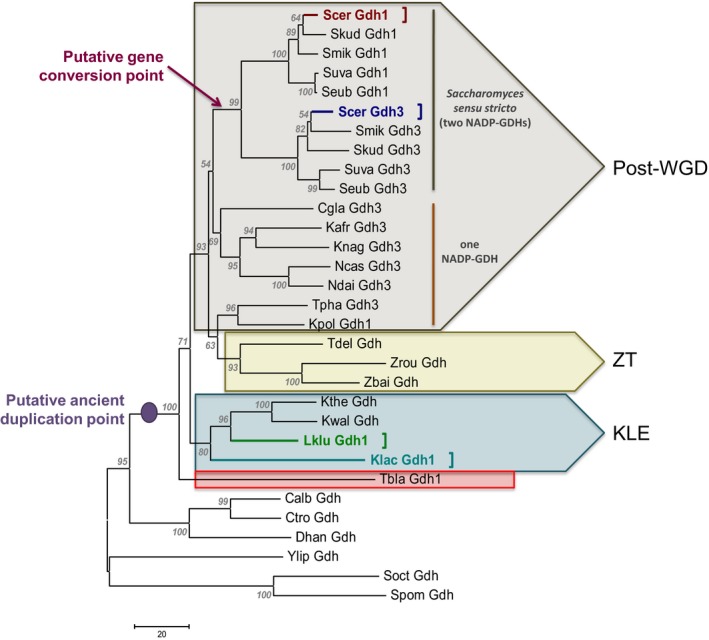
Evolutionary relationships of NADP‐GDHs from yeasts. The phylogeny was constructed using the neighbor‐joining method (Saitou & Nei, 1987). The optimal tree with the sum of branch length = 1043.39892578 is shown. The percentage of replicate trees in which the associated taxa clustered together in the bootstrap test (500 replicates) are shown next to the branches (Felsenstein, 1985). The tree is drawn to scale, with branch lengths in the same units as those of the evolutionary distances used to infer the phylogenetic tree. The evolutionary distances were computed using the number of differences method (Nei & Kumar, 2000) and are in the units of the number of amino acid differences per sequence. The analysis involved 31 amino acid sequences. All ambiguous positions were removed for each sequence pair. There were a total of 484 positions in the final dataset. Evolutionary analyses were conducted in MEGA6 (Tamura et al., 2013). Scer Gdh1, *Saccharomyces cerevisiae Sc*Gdh1 (red letters and square bracket); Scer Gdh3, *Saccharomyces cerevisiae Sc*Gdh3 (dark blue letters and square bracket); Lklu Gdh1, *Lachancea kluyveri Lk*Gdh1 (green letters and square bracket); Klac Gdh1, *Kluyveromyces lactis Kl*Gdh1 (light blue letters and square bracket). Post‐WGD, post‐whole genome duplicacion clade (light brown group); ZT,* Zygosaccharomyces*‐*Torulaspora* clade (light yellow group); KLE,* Kluyveromyces*‐*Lachancea*‐*Eremothecium* clade (light blue group). Tbla Gdh1, *Torulaspora blattae* Gdh1 (red square). NADP‐GDH sequence accession numbers, taxa used and their corresponding abbreviations are included in Table S2

To analyze whether the observed *Sc*Gdh1, *Sc*Gdh3, *Lk*Gdh1, and *Kl*Gdh1 regulatory and kinetic properties correlated with the evolutionary origin of these proteins, a phylogenetic tree was constructed with NADP‐dependent Gdh sequences from fungal representatives of the different taxonomical classes (Figure 6). Overall, GDH phylogenies resembled the taxonomical classifications; however, three major aspects should be highlighted. 1) *Sc*Gdh1 (Scer Gdh1) and *Sc*Gdh3 (Scer Gdh3) were grouped in a separated clade together with Gdh1 and Gdh3 sequences from *Saccharomyces sensu stricto* species but not with other expected orthologs identified by synteny at the YGOB database (e.g., Scer Gdh1 with Lklu Gdh1 or Scer Gdh3 with Cgla Gdh3). These results suggest that gene conversion between the ancestral *GDH* duplicated copies of the *S. cerevisiae* lineage occurred after the whole genome duplication event (Figure 6, red arrow: putative gene conversion point). 2) Aside the *Saccharomyces sensu stricto* clade, all post‐WGD yeasts have conserved only one of the Gdh copies (in almost all of the cases the *Sc*Gdh3 orthologous counterpart) (Figure 6, orange line). 3) *Kl*Gdh1 (Klac Gdh1) and *Lk*Gdh1 (Lklu Gdh1) grouped together and might have been originated from a *GDH* clade which was generated through an ancient genome duplication event that was the consequence of an hybridization process which preceded the divergence between *Saccharomyces* and a clade containing the genera *Kluyveromyces*,* Lachancea* and *Eremothecium* (Figure 6, KLE group), as has been recently proposed by Marcet‐Houben and Gabaldón (2015). This observation is also supported by the fact that *Tetrapisispora blattae* Gdh sequence (Tbla Gdh1) (Figure 6, red rectangle) grouped outside even of the KLE clade and not with the other post‐WGD species *Tetrapisispora phaffii* (Tpha Gdh1) and *Vanderwaltozyma polyspora* (Vpol Gdh1) Gdh sequences as has been previously established (Marcet‐Houben & Gabaldón, 2015).

## 4 Discussion

This study addressed the question of whether there is a correlation between the regulation of the NADP‐GDHs activity and the evolutionary history of the corresponding genes in three *Saccharomycetales* species showing different levels of adaptation to the fermentative lifestyle. To this end, we compared the results of phylogenetic, functional, and kinetic analyses. Presented results show that kinetic properties and heterologous complementation capacities of the *S. cerevisiae Sc*Gdh1 isoform are closer to those of the *L*. *kluyveri Lk*Gdh1 enzyme than to those of its *Sc*Gdh3 paralogous isoform. While kinetics and heterologous complementation abilities of the *Sc*Gdh3 enzyme, resemble those of the *Kl*Gdh1, *K. lactis* enzyme.

### 4.1 Functional characterization was necessary to group the NADP‐GDHs into similar pairs

Carbon source‐dependent transcriptional regulation of the *ScGDH1* gene was similar to that of the *KlGDH1* gene because transcript levels and nucleosome positioning did not change with the nature of the carbon source (Figures 1a, b and 2c and d). Furthermore, transcriptional regulation of the *ScGDH3* and *LkGDH1* genes resulted in higher transcript levels on ethanol than on glucose and this was accompanied by chromatin remodeling (Figures 1a, c and 2a and b). However, kinetic characterization of the NADP‐GDHs and heterologous complementation patterns of the corresponding genes resulted in opposite relationships. Thus, transcriptional responses did not give a complete portrait of Gdh isozymes function. Yet, the close correlation between the NADP‐GDH specific activities (Figure 4) and growth rates (Figure 3) suggest that the *ScGDH1* and the *LkGDH1* genes could be expressed at similar levels in *S. cerevisiae* and in *L. kluyveri*. Similarly, *ScGDH3* and *KlGDH1* genes in *S. cerevisiae* and *K. lactis*, could share similar functions and biological properties. However, further research is necessary to determine the contribution of transcriptional regulation to the heterologous complementation similarities, and to identify the transcriptional regulators involved.

### 4.2 Kinetic properties comparisons allow grouping *Sc*Gdh1 and *Lk*Gdh1 enzymes in a peculiar group, different from *Sc*Gdh3 and *Kl*Gdh1

Hyperbolic kinetics and high affinity for α‐KG are shared properties of the *Sc*Gdh1 and *Lk*Gdh1 enzymes, while cooperativity for α‐KG utilization is a shared property of the *Sc*Gdh3 isoform with the *Kl*Gdh1 enzyme (Table 3, Figure 5). However, the physiological significance of *Sc*Gdh3 cooperativity is not clear since it has been only observed at pH 5.8 (Table 3) or lower (DeLuna et al., 2001). Intracellular pH during exponential growth has been reported to be close to neutrality: 7.2 on glucose and 6.8 on a mix of 2% ethanol and 2% glycerol (Orij, Postmus, Ter Beek, Brul, & Smits, 2009). In non‐growing glucose‐starved cells, however, it drops to 5.5–6.0 (Orij et al., 2009). These last conditions may reflect the stationary phase context in which *Sc*Gdh3 plays a significant role (Lee et al., 2012). It is possible that *Sc*Gdh3 cooperativity is a reminiscent feature of the ancestor protein without a true physiological and metabolic role in vivo; however, it can also be the case that an unknown allosteric effector induces cooperativity during exponential growth on ethanol, whose effect may be mimicked by acidic pH “in vitro*”*. The similarities in complementation patterns of the *Kl*Gdh1 and *Sc*Gdh3 enzymes suggest that cooperativity of *Sc*Gdh3 may be important “in vivo*”*.

Cooperativity for the utilization of the three substrates was observed in the *K. lactis* enzyme *KlGdh1*, most notably for α‐KG and ammonium utilization for which the Hill numbers were 4.4 and 2.7, respectively (Table 3). The estimated α‐KG physiological concentration in this yeast was 0.7 mmol/L (Table 4) and at this concentration, the *Kl*Gdh1 catalytic rate is not highly responsive to changes in α‐KG (Figure 3). This suggests that α‐KG availability does not determine glutamate synthesis in *K. lactis*. However, the low catalytic rate observed for the *Kl*Gdh1 enzyme at 0.7 mmol/L (Figure 3), may not be compatible with the fast growth observed for *K. lactis* (Table 2). It is possible that unknown activators contribute to modulation of the *K. lactis* enzyme in vivo. Interestingly, the *S. cerevisiae* and *L. kluyveri* enzymes are highly responsive to changes in α‐KG at the physiological concentrations (Table 4 and Figure 3) this suggests that the rate of glutamate synthesis is highly influenced by α‐KG availability as was previously proposed (Quezada et al., 2013). Worth of mention is the fact that there is growing evidence indicating that α‐KG plays a role in metabolic regulation. Thus, modulation of the intracellular α‐KG levels could constitute important mechanisms of metabolic control. In this regard, it has been proposed that in *Caenorhabditis elegans*, α‐KG is a key metabolite mediating longevity by dietary restriction (Chin et al., 2014). Intracellular α‐KG/succinate levels can contribute to the maintenance of cellular identity and have a mechanistic role in the transcriptional and epigenetic state of mouse stem cells (Carey, Finley, Cross, Allis, & Thompson, 2015). Most interestingly, recent studies of Gdh1 function has revealed that *gdh1* mutants show enhanced N‐terminal histone H3 proteolisis, suggesting that α−KG has a key regulatory role in telomere silencing in *S. cerevisiae* (Su & Pillus, 2016).

Reported NADPH intracellular concentration is around 286 μmol/L (Zhang et al., 2015; authors considered a cellular volume of 1.7 ml/g cell dry weight), which corresponds to 6–7 *K*_m‐*NADPH*_ or *S*_*0.5‐NADPH*_ (Table 3). At this concentration, the activities of the herein studied NADP‐GDH enzymes were not responsive to changes on the NADPH concentration (Fig. S4). This indicates that the physiological concentration of this substrate is close to saturation and does not determine the NADP‐GDH activity in vivo. By contrast, the reported intracellular ammonium concentration is 2.2 mmol/L (Cueto‐Rojas et al., 2016; considering a cellular volume of 1.7 ml/g cell dry weight as in Zhang et al., 2015). This value is well below the *K*_m‐*NH4+*_ or *S*_*0.5‐NH4+*_ shown in Table 3, which indicates that the ammonium availability modulates the NADP‐GDH activity in vivo. Thus, glutamate synthesis by NADP‐GDH seems to be mainly determined by α‐KG and ammonium availability and not by product inhibition by glutamate.

Most interesting was the fact that, the kinetic behavior of the enzymes present in the two yeast species which show significant fermentative capacity when grown on high glucose media (*Sc*Gdh1 in *S. cerevisiae* and *Lk*Gdh1 in *L. kluyveri*) was hyperbolic, showing high affinity for α‐KG (*K*_m‐α‐KG_ ≈ 0.4 mmol/L). The enzyme present in the yeast with a predominantly respiratory metabolism, *Kl*Gdh1 from *K. lactis*, and the *Sc*Gdh3 isoform whose contribution to glutamate synthesis increases during respiratory metabolism in *S. cerevisiae* (Table 2), were cooperative and showed low affinity for α‐KG (*S*_0.5*Kl*Gdh1, pH7.5_ = 3.61 mmol/L and *S*_0.5*Sc*Gdh3, pH5.8_ = 1.95 mmol/L). Assuming that during *S. cerevisiae* evolution, *L. kluyveri* and *K. lactis*, selective pressures drove changes in the NADP‐GDHs, these enzymes could have changed from cooperative to hyperbolic. As cooperativity was observed in the NADP‐GDH from *K. lactis* and in the *Sc*Gdh3 isoform, it seems possible that the common ancestor of the three yeast species had a cooperative NADP‐GDH and that this property was lost two times: one in the *L. kluyveri* lineage after divergence of the *S. cerevisiae* and *L. kluyveri* branches, and the other after the WGD event which resulted in the conservation of the hyperbolic *Sc*Gdh1 and the cooperative *Sc*Gdh3. This suggests that NADP‐GDH kinetics may be related to adaptation to the fermentative or respiratory lifestyles, and further research in various yeast species is needed to explore this possibility.

## Conflict of Interest

None declared.
