# Supplementary material for: Diversification of the kinetic properties of yeast NADP‐glutamate‐dehydrogenase isozymes proceeds independently of their evolutionary origin
Source: Microbiologyopen. 2016 Nov 19;6(2):e00419. doi: 10.1002/mbo3.419 (PMC5387307; doi:10.1002/mbo3.419)
Supplement: Supplementary file 1 [file MBO3-6-na-s001.doc]

**Fig. S1**. Glutamic Inhibition of NADP-GDHs


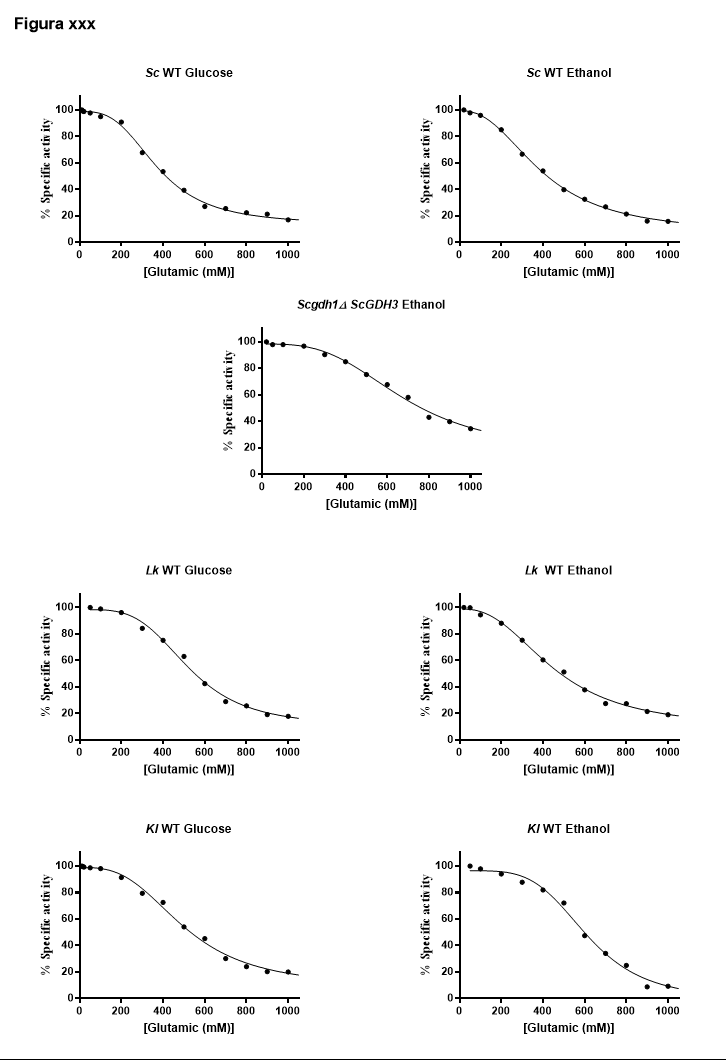


|  | ***Sc* WT Glucose** | ***Sc* WT Ethanol** | ***Scgdh1∆* WT Ethanol** | ***Lk* WT Glucose** | ***Lk* WT Ethanol** | ***Kl* WT Glucose** | ***Kl* WT Ethanol** |
| --- | --- | --- | --- | --- | --- | --- | --- |
| **IC_50_** | 376 ± 10.3 | 397.7 ± 9.9 | 681.3 ± 17.5 | 523.7 ± 18.8 | 454.6 ±20.1 | 503 ± 22.9 | 611.6 ± 24.7 |

Glutamic Inhibition of the NADP-GDH activity in clarified extracts from *Sc* WT*, Scgdh1Δ, Lk* WT and *Kl* WT are shown. Cells were cultured on MM with ammonium sulfate as nitrogen source and 2% glucose or ethanol as carbon source. Experimental data were fitted with GraphPad Prism 7.00 (Software Inc.), the R^2^ were higher than 0.993

**Fig. S2**. NADP-GDH purification

**
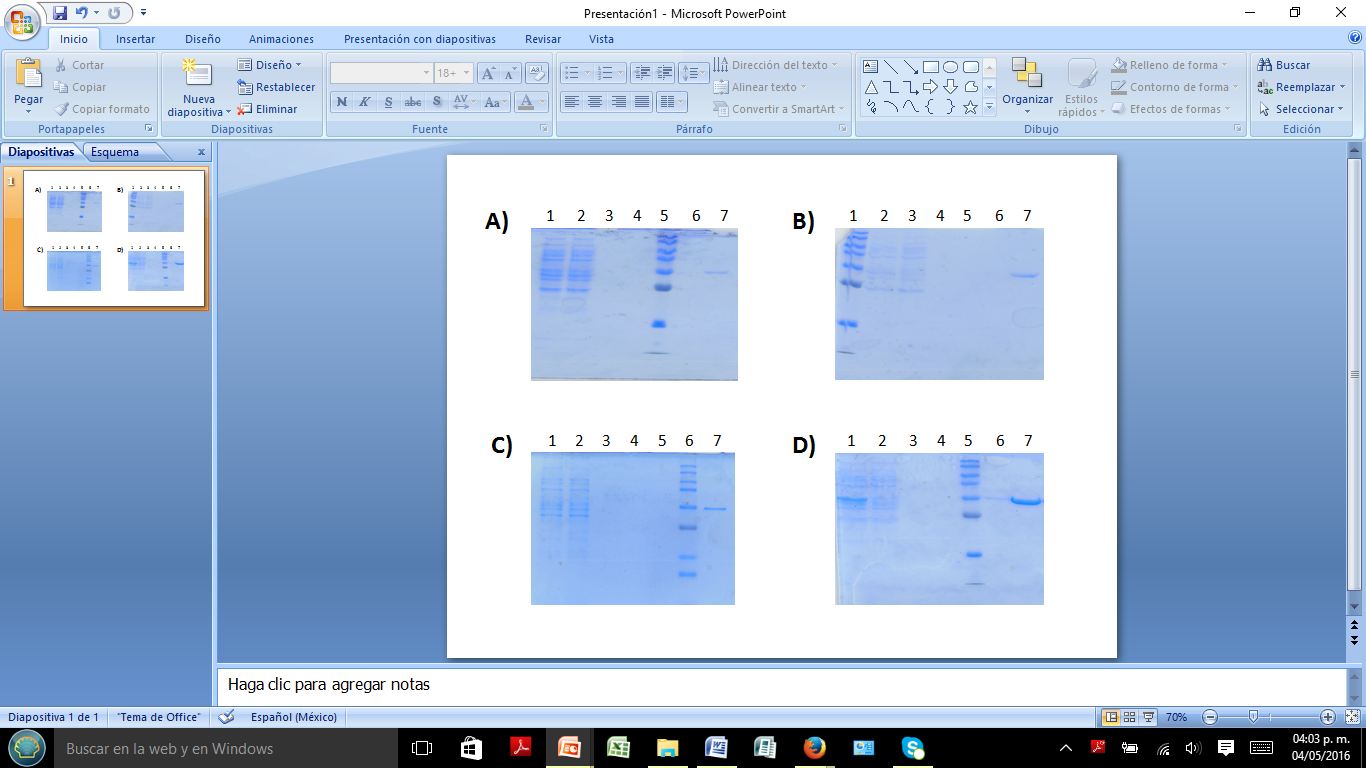
**

Recombinant enzymes from *S. cerevisiae, L. kluyveri* and *K. lactis* were produced in *E. coli* as described in the Experimental procedures section. **A)** *Sc*Gdh1 (53 kD): Lane 1,soluble fraction; Lane 2, not attached fraction; Lane 3, washing 30 mM imidazol; Lane 4, washing 40 mM imidazol; Lane 5, Ladder precision plus protein broad range; Lane 6, washing 50 mM imidazol and Lane 7, elution 500 mM imidazol. **B)** *Sc*Gdh3 (55 kD): Lane 1, Ladder precision plus protein broad range; Lane 2, soluble fraction; Lane 3, not attached fraction; Lane 4, washing 30 mM imidazol; Lane 5, washing 40 mM imidazol; Lane 6, washing 50 mM imidazol and Lane 7, elution 500 mM imidazol. **C)** *Lk*Gdh1 (54 kD): Lane 1,soluble fraction; Lane 2, not attached fraction; Lane 3, washing 30 mM imidazol; Lane 4, washing 40 mM imidazol; Lane 5,washing 50 mM imidazol; Lane 6, Ladder precision plus protein broad range and Lane 7, elution 500 mM imidazol. And **D)** *Kl*Gdh1 (54 kD): Lane 1,soluble fraction; Lane 2, not attached fraction; Lane 3, washing 30 mM imidazol; Lane 4, washing 40 mM imidazol; Lane 5, Ladder precision plus protein broad range; Lane 6, washing 50 mM imidazol and Lane 7, elution 500 mM imidazol.

**Fig. S3.** NADP-GDH kinetic responses to NADPH saturation

**
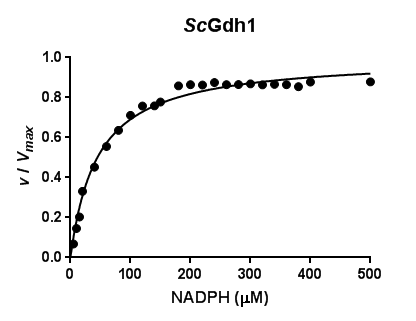

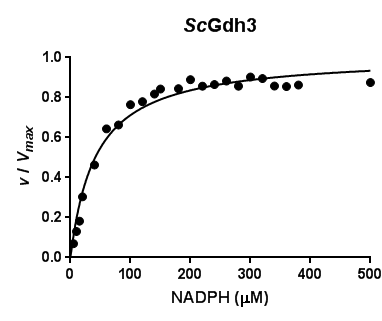

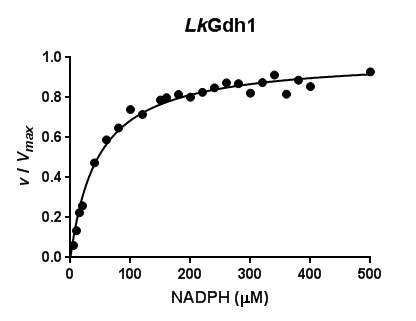

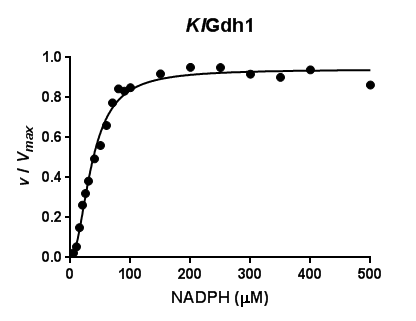
**

Initial velocities are shown as fractions of the corresponding *V_max_* at different NADPH concentrations. The reductive amination reaction was measured at pH 7.5 using pure recombinant proteins from *S. cerevisiae* (*Sc*Gdh1 and *Sc*Gdh3), *L. kluyveri* (*Lk*Gdh1) and *K. lactis* (*Kl*Gdh1). The corresponding kinetic parameters are shown in Table 3.

**Fig. S4**. NADP-GDH kinetic responses to ammonium saturation

**
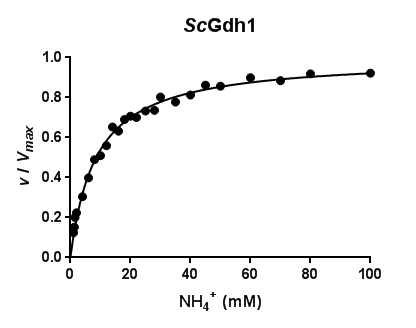

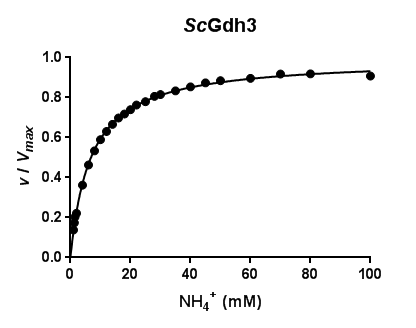

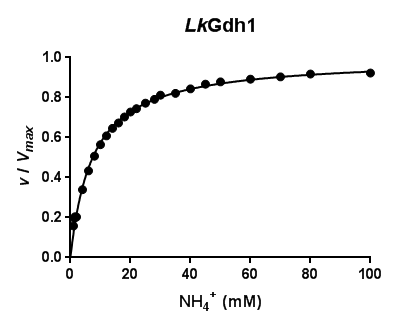

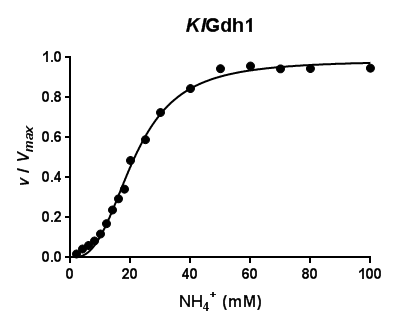
**

Initial velocities are shown as fractions of the corresponding *V_max_* at different ammonium (NH_4_^+^) concentrations. The reductive amination reaction was measured at pH 7.5 using pure recombinant proteins from *S. cerevisiae* (*Sc*Gdh1 and *Sc*Gdh3), *L. kluyveri* (*Lk*Gdh1) and *K. lactis* (*Kl*Gdh1). The corresponding kinetic parameters are shown in Table 3.

**Table S1.** Oligonucleotides used in the present work

| **Primer** | **Oligonucleotide sequence (5’- 3’) *** | **Sequences in bold letters** |
| --- | --- | --- |
| 101 | AACTGGAATTACACCCTTGGCTGAC |  |
| 102 | **CCAGTGTCGAAAACGAGCTCG**ATTGTGCGTATCTACAGATGCTTGTGG | 21bp homologous to the 5’ end of the *kan*MX4 selection marker |
| 103 | **CTGGCGCGCCTTAATTAACCCGAG**GTGACGTCTGGGAATAAGCTCC | 24bp homologous to the 3’ end of the *kan*MX4 selection marker |
| 104 | CTCTACCGTTGGTCTTGGTATGTTTG |  |
| 105 | CGGGTTAATTAAGGCGCGCCAG |  |
| 106 | CGAGCTCGTTTTCGACACTGG |  |
| 107 | GCACCAATGCCACTCTCTTTTCTTCGG |  |
| 108 | GGAGTTAAACTACAGGACCCGGAG |  |
| 108-1 | CCTCGTTGGCTGTTTCAGATGG |  |
| 108-2 | CTGCAGCGAGGAGCCGTAAT |  |
| 109 | CGCTCCGGCGATATGCTATCACACGGCTCATCATGGCGGATTCCGCTGGTATATAAGACC**CTCCTTGACAGTCTTGACGTGC** | 22bp homologous to the 5’ end of the *nat*MX4 selection marker |
| 110 | **AGAAAAAAACAAACTTGAAGCAG**AGTCACGTCTTCAATGCCTATCTACTGACAGTGCACCGACAGCAGTATAGCGACCAGC | 23bp homologous to the 3’ end of the *nat*MX4 selection marker |
| 111 | CCTCTTCATCATCCGCAGATAA |  |
| 112 | GCACGTCAAGACTGTCAAGGAGGGTCTTATATACCAGCGGAATC |  |
| 113 | GCTGGTCGCTATACTGCTGTCGGTGCACTGTCAGTAGATAGGC |  |
| 114 | GCCTATTCCTGCATCCTTTGA |  |
| 115 | GTAGTCGTCTTGTGACTTGGC |  |
| 116 | TTCCTCGTCGTCACCAGCAT |  |
| 117 | GATTGATTATTTGTGAAAAGTAATAAGAACTAATACTATACCAAAACTTTTAAAG**AGATCTGTTTAGCTTGCCTCGTCCCCGCCG** | 30bp homologous to the 5’ end of the *nat*MX4 selection marker |
| 118 | CGGAATTAAAGTTCATTTTTAAATTTATGCTTTTCTAAGATAAGAACACTCACTGAATT**CGAGCTCGTTTTCGACACTGGATGG** | 25 bp homologous to the 3’ end of the *nat*MX4 selection marker |
| 119 | ATGAGAAAAGATAAAGACTGAATACCAACCCAAAAGGACAGTTTCGAGCGTATCACGCAA |  |
| 120 | **CCGGGTGACCCGGCGGGGACGAGGCAAGCTAAACAGATCT**CTTTAAAAGTTTTGGTATAGTATTAGTTC | 40bp homologous to the 5’ end of the *nat*MX4 selection marker |
| 121 | **ACTAACGCCGCCATCCAGTGTCGAAAACGAGCTCGAATTC**AGTGAGTGTTCTTATCTTAGAAAAGC | 40bp homologous to the 3’ end of the *nat*MX4 selection marker |
| 122 | TATTGAACTTGGTATCTTCGCTTTCCACCTGAGAAATCTTCTTGCCGACTTTACCAT |  |
| 123 | GTGGCTATCTTGACGTCTGGCAGTTATACAG |  |
| 124 | CAGAAACAGGTTGGCCTTACAAATGCAAGC |  |
| 125 | GCGCGC**GCTAGC**ATGTCAGAGCCAGAATTTCAACAAGC | *Nhe*I restriction site |
| 126 | GCGCGC**CTCGAG**TTAAAATACATCACCTTGGTC | *Xho*I restriction site |
| 127 | GCGCGC**GCTAGC**ATGACAAGCGAACCAGAGTTTCAGC | *Nhe*I restriction site |
| 128 | GCGCGC**CTCGAG**TAAAAAACGTCTCCCTGGTCAAG | *Xho*I restriction site |
| 129 | GCGCGC**CATATG**ATGGCCCAACCATACGAACC | *Nde*I restriction site |
| 130 | GCGCGC**GGATCC**TTATTCCCAGACGTCACCTTG | *Bam*HI restriction site |
| 131 | GCGCGC**GCTAGC**ATGTCTGCTGAACCATATGAACC | *Nhe*I restriction site |
| 132 | GCGCGC**GGATCC**TTAGAAAACATCACCTTGGTGC | *Bam*HI restriction site |
| 133 | GCGCGC**GGATCC**CTTTAGGTTCAAGTCCGCTAAACTCTACA | *Bam*HI restriction site |
| 134 | CGCGCG**CTCGAG**ATTCCTTACCGTTTGTGGCATAGTCGATCA | *Xho*I restriction site |
| 135 | GCGCGC**CTCGAG**TAATGTTAAGATGAAATTTAAGTGAGCTGG | *Xho*I restriction site |
| 136 | CGCGC**GGATCC**ACACGTTTGCCCTCAAACGACTCCTTGCCG | *Bam*HI restriction site |
| 137 | GCGCGC**GGATCC**GGTTGATTTACTTTTGAAATGGTCCTTCCT | *Bam*HI restriction site |
| 138 | CGCGCG**GAGCTC**GGAGCTTATTCCCAGACGTCAC | *Sac*I restriction site |
| 139 | GCGCGC**GGATCC**ACCATAGCGTTTTTCGCCGTCCGCTG | *Bam*HI restriction site |
| 140 | CGCGCG**TCTAGA**CATGGATTTCAAATCCTAAATTTATATAAC | *Xba*I restriction site |
| 141 | GAGAATTGAGCAGACACATT |  |
| 142 | AATAGCTTCTGGAGTGGAA |  |
| 143 | AGTGTTACCTATTGTTTCTGTCCCGGAG |  |
| 144 | CTGTATGCAGTCGTTGAAGCAGTTGATC |  |
| 145 | GTTTTGCCGGTGACGAC |  |
| 146 | CTTTCGGCAATACCTGGG |  |
| 147 | ATGGCCCAACCATACGAACCAGAATTTC |  |
| 148 | GAGAGTTTTGAGCCATTTCCAAGCCAG |  |
| 149 | AAGCTCGTAGTTGAACTTTGGGTC |  |
| 150 | AAGACTTTGATTTCTCGTAAGGTGCCG |  |
| 151 | ATGTCTGCTG AACCATATGA ACC |  |
| 152 | TTAGAAAACATCACCTTGGTGC |  |
| 153 | AAGCTCGTAGTTGAACTTTGGGTCTGG |  |
| 154 | AAGACTTTGATTTCTCGTAAGGTGCCG |  |

*All oligonucleotides were generated in this work.

**Table S2.** Sequence accession numbers

|  | **Specie** | **Abbreviation (phylogenetic tree)** | **Systematic gene name or Accession number (NCBI)** |
| --- | --- | --- | --- |
| **Post-WGD** | *Saccharomyces cerevisiae* | Scer Gdh1 | YOR375C |
|  | *Saccharomyces kudriavzevii* | Skud Gdh1 | EJT43926 |
|  | *Saccharomyces mikatae* | Smik Gdh1 | Smik_c510_20577 |
|  | *Saccharomyces uvarum* | Suva Gdh1 | Suva_c773_24096 |
|  | *Saccharomyces eubayanus* | Seub Gdh1 | KOG96846 |
|  | *Saccharomyces cerevisiae* | Scer Gdh3 | YAL062W |
|  | *Saccharomyces kudriavzevii* | Skud Gdh3 | EJT44409 |
|  | *Saccharomyces mikatae* | Smik Gdh3 | Smik_c1235_25 |
|  | *Saccharomyces uvarum* | Suva Gdh3 | Suva_c942_30 |
|  | *Saccharomyces eubayanus* | Seub Gdh3 | KOH01335 |
|  | *Candida glabrata* | Cgla Gdh3 | CAGL0D00176g |
|  | *Kazachstania africana* | Kafr Gdh3 | KAFR0I00150 |
|  | *Kazachstania naganishii* | Knag Gdh3 | KNAG0E04210 |
|  | *Naumovozyma castellii* | Ncas Gdh3 | NCAS0A07680 |
|  | *Naumovozyma dairenensis* | Ndai Gdh3 | NDAI0H02070 |
|  | *Tetrapisispora phaffii* | Tpha Gdh3 | TPHA0N00160 |
|  | *Vanderwaltozyma polyspora* | Kpol Gdh1 | Kpol_538.50 |
| **ZT** | *Torulaspora delbrueckii* | Tdel Gdh | TDEL0H04470 |
|  | *Zygosaccharomyces rouxii* | Zrou Gdh | ZYRO0C00396g |
|  | *Zygosaccharomyces bailii* | Zbai Gdh | CDH13190 |
| **KLE** | *Lachancea thermotolerans* | Kthe Gdh | KLTH0D00550g |
|  | *Kluyveromyces waltii* | Kwal Gdh | Kwal_26.6727 |
|  | *Lachancea kluyveri* | Lklu Gdh1 | SAKL0D14982g |
|  | *Kluyveromyces lactis* | Klac Gdh1 | KLLA0F00594g |
|  | *Tetrapisispora blattae* | Tbla Gdh1 | TBLA0F01800 |
|  | *Candida albicans* | Calb Gdh | EAK91047 |
|  | *Candida tropicalis* | Ctro Gdh | EER32364 |
|  | *Debaryomyces hansenii* | Dhan Gdh | CAG86519 |
|  | *Yarrowia lipolytica* | Ylip Gdh | CAG78362 |
|  | *Schizosaccharomyces octosporus* | Soct Gdh | EPX72089 |
|  | *Schizosaccharomyces pombe* | Spom Gdh | SPCC622.12c.1 |

**Table S3.** Primers used for nucleosome scanning assays in A) *ScGDH1,* B) *ScGDH3,* C) *LkGDH1* and D) *KlGDH1* locus

A)

| **Primer** | **Sequence** | **Middle of amplicon (Promoter coordinate)** | **5’/3’ end** | **Size (bp)** |
| --- | --- | --- | --- | --- |
| **1***-****ScGDH1*** | CGACAAGAAGGAGATGAACTT | -820 | -871 | 103 |
| **1***-* ***ScGDH1*** | CCACAGCCCGCTAGAATAATT |  | -768 |  |
| **2***-* ***ScGDH1*** | CAGTGATTCTGTCCAGCATTG | -769 | -823 | 108 |
| **2***-* ***ScGDH1*** | CACTTTATACTGAATGGAGTTACT |  | -715 |  |
| ***3- ScGDH1*** | AATTATTCTAGCGGGCTGTGG | -744 | -798 | 109 |
| **3***-* ***ScGDH1*** | TTATCGCAGCCCCATGAAG |  | -689 |  |
| **4***-* ***ScGDH1*** | AGTAACTCCATTCAGTATAAAGTG | -690 | -739 | 99 |
| **4***-* ***ScGDH1*** | ATGCGGAGTGGTGCCCA |  | -640 |  |
| **5***-* ***ScGDH1*** | CTTCATGGGGCTGCGATAAA | -664 | -709 | 91 |
| **5***-* ***ScGDH1*** | GTATAATTCAGGTTATGCCCAG |  | -618 |  |
| **6***-* ***ScGDH1*** | TGGGCACCACTCCGCAT | -616 | -657 | 83 |
| **6***-* ***ScGDH1*** | TTATCCAGCCAATCGTAAACG |  | -574 |  |
| **7***-* ***ScGDH1*** | CTGGGCATAACCTGAATTATAC | -589 | -640 | 103 |
| **7***-* ***ScGDH1*** | GAGTGGATGTAGCATCATATTC |  | -537 |  |
| **8***-* ***ScGDH1*** | CGTTTACGATTGGCTGGATAA | -549 | -595 | 93 |
| **8***-* ***ScGDH1*** | CATAAGGGGAGCCTGATACA |  | -502 |  |
| **9***-* ***ScGDH1*** | AATATGATGCTACATCCACTCA | -512 | 559 | 94 |
| **9***-* ***ScGDH1*** | TAAGATCAGGCCCGTTTCCA |  | -465 |  |
| **10***-* ***ScGDH1*** | TGTATCAGGCTCCCCTTATG | -475 | -521 | 92 |
| **10***-* ***ScGDH1*** | TCGAGGCCATCCAATCAGA |  | -429 |  |
| **11***-* ***ScGDH1*** | TGGAAACGGGCCTGATCTTA | -432 | -484 | 104 |
| **11***-* ***ScGDH1*** | TGAAAATGCATGGGCCGGTT |  | -380 |  |
| **12***-* ***ScGDH1*** | ATCTGATTGGATGGCCTCGA | -391 | -448 | 114 |
| **12***-* ***ScGDH1*** | ACGTGGGGTCGTACTATTTC |  | -334 |  |
| **13***-* ***ScGDH1*** | AACCGGCCCATGCATTTTCA | -355 | -400 | 91 |
| **13***-* ***ScGDH1*** | AGCTGATAACAGCTTCTCTCT |  | -309 |  |
| **14***-* ***ScGDH1*** | GAAATAGTACGACCCCACGT | -311 | -359 | 96 |
| **14***-* ***ScGDH1*** | TGCTGATTTTCATTATGGTACCT |  | -263 |  |
| **15***-* ***ScGDH1*** | AGAGAGAAGCTGTTATCAGCT | -279 | -330 | 103 |
| **15***-* ***ScGDH1*** | CTACTTCTTACGCTTTCTTCTTC |  | -227 |  |
| **16***-* ***ScGDH1*** | AGGTACCATAATGAAAATCAGCA | -242 | -286 | 88 |
| **16***-* ***ScGDH1*** | TACGTATACTTTGCTTTAACAAGAA |  | -198 |  |
| **17***-* ***ScGDH1*** | GAAGAAGAAAGCGTAAGAAGTAG | -204 | -250 | 92 |
| **17***-* ***ScGDH1*** | GAAAATTTTCCAATCTTCTCTTACTT |  | -158 |  |
| **18***-* ***ScGDH1*** | TTCTTGTTAAAGCAAAGTATACGTA | -162 | -213 | 103 |
| **18***-* ***ScGDH1*** | ATGGGTAAACGCATTTGTAACTC |  | -110 |  |
| **19***-* ***ScGDH1*** | AAGTAAGAGAAGATTGGAAAATTTTC | -131 | -184 | 106 |
| **19- *ScGDH1*** | GAAAAGTCATTTAAAGAGTGAGAG |  | -78 |  |
| **20***-* ***ScGDH1*** | GAGTTACAAATGCGTTTACCCAT | -86 | -133 | 95 |
| **20***-* ***ScGDH1*** | TATATTAGAATAATGCGATAGTACGT |  | -38 |  |
| **21***-* ***ScGDH1*** | CTCTCACTCTTTAAATGACTTTTC | -51 | -100 | 98 |
| **21***-* ***ScGDH1*** | TCTTTTTCTTTTTGGTCTCCTAAC |  | -2 |  |
| **22***-* ***ScGDH1*** | ACGTACTATCGCATTATTCTAATATA | +3 | -50 | 94 |
| **22***-* ***ScGDH1*** | GAGACAACTTCTTCGTAAGCT T |  | +44 |  |
| **23***-* ***ScGDH1*** | GTTAGGAGACCAAAAAGAAAAAGA | +9 | -25 | 103 |
| **23***-* ***ScGDH1*** | GTGTTGTTCGAAAAGAGTAGAGT |  | +78 |  |
| **24***-* ***ScGDH1*** | AAGCTTACGAAGAAGTTGTCTC | +27 | +22 | 96 |
| **24***-* ***ScGDH1*** | CTGGAACAGAAACAATTGGCAA |  | +118 |  |
| **25***-* ***ScGDH1*** | ACTCTACTCTTTTCGAACAACAC | +110 | +55 | 99 |
| **25***-* ***ScGDH1*** | TCATTTTCCCAGGTGACTCTG |  | +154 |  |
| **26***-* ***ScGDH1*** | TTGCCAATTGTTTCTGTTCCAG | +148 | +96 | 104 |
| **26***-* ***ScGDH1*** | TTATATTGCACTCTGTAACCTTGA |  | +200 |  |
| **27***-* ***ScGDH1*** | CAGAGTCACCTGGGAAAATG | +183 | +134 | 97 |
| **27***-* ***ScGDH1*** | TAGACCACCCTTGTATGGAC |  | +231 |  |
| **28***-* ***ScGDH1*** | TCAAGGTTACAGAGTGCAATATAA | +225 | +176 | 97 |
| **28***-* ***ScGDH1*** | CAAGAATTTCAAGATAGACAAGTTC |  | +273 |  |

B)

| **Primer** | **Sequence** | **Middle of amplicon (Promoter coordinate)** | **5’/3’ end** | **Size (bp)** |
| --- | --- | --- | --- | --- |
| **1***-****ScGDH3*** | TGACGCACAAGATTCATAACAAAT | -826 | -877 | 103 |
| **1***-* ***ScGDH3*** | ACATTAAAAATATTTACAGCCTAGCTT |  | -774 |  |
| **2***-* ***ScGDH3*** | TACATTGTGCAGAAGGTCTTCA | -783 | -832 | 98 |
| **2***-* ***ScGDH3*** | AACAAATTATGCCTCACTTGATATTA |  | -734 |  |
| ***3- ScGDH3*** | AAGCTAGGCTGTAAATATTTTAATGT | -754 | -800 | 93 |
| **3***-* ***ScGDH3*** | GAAGTTCAGCTACATATAACAAATTA |  | -707 |  |
| **4***-* ***ScGDH3*** | TAATATCAAGTGAGGCATAATTTGTT | -715 | -750 | 70 |
| **4***-* ***ScGDH3*** | CTGAAACAATCCGGTGCTTG |  | -680 |  |
| **5***-* ***ScGDH3*** | TAATTTGTTATATGTAGCTGA ACTTC | -684 | -733 | 99 |
| **5***-* ***ScGDH3*** | GAATAATAGCTTCTACACTTTGAATT |  | -634 |  |
| **6***-* ***ScGDH3*** | CAAGCACCGGATTGTTTCAG | -629 | -680 | 103 |
| **6***-* ***ScGDH3*** | ACAAGCTGCCACAAGTATGTTTA |  | -577 |  |
| **7***-* ***ScGDH3*** | AATTCAAAGTGTAGAAGCTATTATTC | -602 | -660 | 116 |
| **7***-* ***ScGDH3*** | GACCCAACAAAACTTAAAAATAAAAC |  | -544 |  |
| **8***-* ***ScGDH3*** | AACATACTTGTGGCAGCTTGT | -549 | -598 | 98 |
| **8***-* ***ScGDH3*** | CGTTTTATCATACTTTACTTTTTCTTT |  | -500 |  |
| **9***-* ***ScGDH3*** | GTTTTATTTTTAAGTTTTGTTGGGTC | -526 | -570 | 89 |
| **9***-* ***ScGDH3*** | ATGGGCGTTAATTACTTTGGCA |  | -481 |  |
| **10***-* ***ScGDH3*** | AAAGAAAAAGTAAAGTATGATAAAACG | -486 | -527 | 82 |
| **10***-* ***ScGDH3*** | TATATGCCTCCTATGCCTTCTT |  | -445 |  |
| **11***-* ***ScGDH3*** | TGCCAAAGTAATTAACGCCCAT | -447 | -493 | 92 |
| **11***-* ***ScGDH3*** | AGAATATCTGTCAGCAGCCATA |  | -401 |  |
| **12***-* ***ScGDH3*** | AAGAAGGCATAGGAGGCATATA | -412 | -467 | 110 |
| **12***-* ***ScGDH3*** | GAAGAAAAAGAAAAGTTGGTATAATAT |  | -357 |  |
| **13***-* ***ScGDH3*** | TATGGCTGCTGACAGATATTCT | -372 | -423 | 103 |
| **13***-* ***ScGDH3*** | TCTTCAAAGAGCTGGGCCAA |  | -320 |  |
| **14***-* ***ScGDH3*** | ATATTATACCAACTTTTCTTTTTCTTC | -332 | -384 | 104 |
| **14***-* ***ScGDH3*** | CTTTAAAATCTCATTGGCTCCCT |  | -280 |  |
| **15***-* ***ScGDH3*** | TTGGCCCAGCTCTTTGAAGA | -285 | -340 | 110 |
| **15***-* ***ScGDH3*** | ACTGTCCCTTTAATATCAATACTG |  | -230 |  |
| **16***-* ***ScGDH3*** | AGGGAGCCAATGAGATTTTAAAG | -251 | -303 | 104 |
| **16***-* ***ScGDH3*** | TGGTCATCACTTTTTCCATATTAAC |  | -199 |  |
| **17***-* ***ScGDH3*** | CAGTATTGATATTAAAGGGAAGT | -202 | -254 | 104 |
| **17***-* ***ScGDH3*** | GTGAAAGTGAAATAAAAAGAAATACTC |  | -150 |  |
| **18***-* ***ScGDH3*** | GTTGGTTAATATGGAAAAAGTGATG | -173 | -224 | 102 |
| **18***-* ***ScGDH3*** | TCAAAGTCAGAAGTCATTAACTGT |  | -122 |  |
| **19***-* ***ScGDH3*** | GAGTATTTCTTTTTATTTCACTTTCAC | -128 | -177 | 99 |
| **19- *ScGDH3*** | GTGTGGCCTATGTATGTACCT |  | -78 |  |
| **20***-* ***ScGDH3*** | AGGTACATACATAGGCCACAC | -54 | -99 | 91 |
| **20***-* ***ScGDH3*** | GTTCGCTTGTCATTTTTTACTTTTTT |  | +8 |  |
| **21***-* ***ScGDH3*** | ATATAGGGAAGTAGCAACAGTCA | -5 | -50 | 91 |
| **21***-* ***ScGDH3*** | ATCTCATCGTAAGCCTGCTGA |  | +41 |  |
| **22***-* ***ScGDH3*** | AAAAAGTAAAAAATGACAAGCGAAC | -43 | -12 | 109 |
| **22***-* ***ScGDH3*** | CTTTTTTATACTGTGGGAATTTTTCAA |  | +97 |  |
| **23***-* ***ScGDH3*** | TCAGCAGGCTTACGATGAGAT | +71 | +20 | 102 |
| **23***-* ***ScGDH3*** | TCCGGGACAGAAACAATAGGT |  | +122 |  |
| **24***-* ***ScGDH3*** | TTGAAAAATTCCCACAGTATAAAAAAG | +121 | +70 | 102 |
| **24***-* ***ScGDH3*** | CTTGCTCGCCATTATCATTTTC |  | -172 |  |
| **25***-* ***ScGDH3*** | ACCTATTGTTTCTGTCCCGGA | +151 | +101 | 99 |
| **25***-* ***ScGDH3*** | AACTGCACCCTGTATCCTTGA |  | +200 |  |
| **26***-* ***ScGDH3*** | GAAAATGATAATGGCGAGCAAG | +200 | +150 | 100 |
| **26***-* ***ScGDH3*** | CTGATGGGTGGAAGCGTAG |  | +250 |  |
| **27***-* ***ScGDH3*** | TCAAGGATACAGGGTGCAGTT | +176 | +129 | 94 |
| **27***-* ***ScGDH3*** | AAATTTTAGGATAGACAGGTTCAC |  | +223 |  |
| **28***-* ***ScGDH3*** | CATAGGCCACACACACACA | -30 | -90 | 121 |
| **28***-* ***ScGDH3*** | AAGCCTGCTGAAACTCTGGT |  | +31 |  |
| **29***-* ***ScGDH3*** | TAGCAACAGTCACCGAAAAGAA | -6 | -39 | 90 |
| **29***-* ***ScGDH3*** | AATCCTCCACAGAAGAAACGA T |  | +51 |  |

**C)**

| **Primer** | **Sequence** | **Middle of amplicon (Promoter coordinate)** | **5’/3’ end** | **Size (bp)** |
| --- | --- | --- | --- | --- |
| **1***-****LkGDH1*** | GTTGGAATCAAAAACTGGCATCA | -895 | -946 | 102 |
| **1***-****LkGDH1*** | TGATCCTTTCCACTTTGTCACT |  | -844 |  |
| **2***-****LkGDH1*** | ACTTTTGAAATGGTCCTTCCTG | -863 | -911 | 97 |
| **2***-****LkGDH1*** | TTGCATCTGGTAGGACGTTCA |  | -814 |  |
| ***3-LkGDH1*** | AGTGACAAAGTGGAAAGGATCA | -815 | -865 | 101 |
| **3***-****LkGDH1*** | AACCTTCCATGTTTCGTCATC |  | -764 |  |
| **4***-****LkGDH1*** | TGAACGTCCTACCAGATGCAA | -778 | -834 | 113 |
| **4***-****LkGDH1*** | TGTTATCCCACGTGAGTACTT |  | -721 |  |
| **5***-****LkGDH1*** | GATGACGAAACATGGAAGGTT | -738 | -784 | 92 |
| **5***-****LkGDH1*** | GTTCTATTTAGCATATGTAGTTGG |  | -692 |  |
| **6***-****LkGDH1*** | AAGTACTCACGTGGGATAACA | -695 | -741 | 93 |
| **6***-****LkGDH1*** | AGTAAGGGACTTGGAGCTTG |  | -648 |  |
| **7***-****LkGDH1*** | CCAACTACATATGCTAAATAGAAC | -664 | -715 | 103 |
| **7***-****LkGDH1*** | GAGGAAGGAGTCGAAAAAGAA |  | -612 |  |
| **8***-****LkGDH1*** | CAAGCTCCAAGTCCCTTACT | -620 | -667 | 95 |
| **8***-****LkGDH1*** | AGGGTTTTTTCAGTCCACGAA |  | -572 |  |
| **9***-****LkGDH1*** | TTCTTTTTCGACTCCTTCCTC | -590 | -632 | 85 |
| **9***-****LkGDH1*** | ACGCCCTGTAAATGGCATCTT |  | -547 |  |
| **10***-****LkGDH1*** | TTCGTGGACTGAAAAAACCCT | -558 | -592 | 69 |
| **10***-****LkGDH1*** | GTTTTATGAAAGAGGTGCCATTT |  | -523 |  |
| **11***-****LkGDH1*** | AAGATGCCATTTACAGGGCGT | -511 | -567 | 112 |
| **11***-****LkGDH1*** | GAGTGCTTAATTCTGACCAATC |  | -455 |  |
| **12***-****LkGDH1*** | AAATGGCACCTCTTTCATAAAAC | -473 | -523 | 100 |
| **12***-****LkGDH1*** | GTGGAGGGCACACAAAATTG |  | -423 |  |
| **13***-****LkGDH1*** | GATTGGTCAGAATTAAGCACTC | -424 | -476 | 105 |
| **13***-****LkGDH1*** | AACGTCGCCTCGGTTTTCTC |  | -371 |  |
| **14***-****LkGDH1*** | CAATTTTGTGTGCCCTCCAC | -392 | -442 | 101 |
| **14***-****LkGDH1*** | TACTGCGAAAAAGGCGTGCT |  | -341 |  |
| **15***-****LkGDH1*** | GAGAAAACCGAGGCGACGTT | -336 | -390 | 109 |
| **15***-****LkGDH1*** | AATCGGAACGTTTCGTCGCC |  | -281 |  |
| **16***-****LkGDH1*** | AGCACGCCTTTTTCGCAGTA | -309 | -360 | 102 |
| **16***-****LkGDH1*** | AACGCACTTGTCCTAAGCCA |  | -258 |  |
| **17***-****LkGDH1*** | GGCGACGAAACGTTCCGATT | -251 | -300 | 98 |
| **17***-****LkGDH1*** | AGCAAGATGAATCCAATCAACG |  | -202 |  |
| **18***-****LkGDH1*** | TGGCTTAGGACAAGTGCGTT | -224 | -277 | 106 |
| **18***-****LkGDH1*** | CTATCTTGTCAAGCCTGCGT |  | -171 |  |
| **19***-****LkGDH1*** | CGTTGATTGGATTCATCTTGCT | -202 | -223 | 103 |
| **19***-****LkGDH1*** | TACGCTCTACACCAAATCAAC |  | -120 |  |
| **20***-****LkGDH1*** | ACGCAGGCTTGACAAGATAG | -141 | -191 | 100 |
| **20***-****LkGDH1*** | CCTGGGGTATTTATACGTTTTAG |  | -91 |  |
| **21***-****LkGDH1*** | GTTGATTTGGTGTAGAGCGTA | -91 | -140 | 99 |
| **21***-****LkGDH1*** | AAGAGACAAAAACCTACAAAAACC |  | -41 |  |
| **22***-****LkGDH1*** | CTAAAACGTATAAATACCCCAGG | -62 | -113 | 102 |
| **22***-****LkGDH1*** | GTGCGTATCTACAGATGCTTG |  | -11 |  |
| **23***-****LkGDH1*** | GGTTTTTGTAGGTTTTTGTCTCTT | +8 | -64 | 112 |
| **23***-****LkGDH1*** | GACTTCGTTGTAAGCTTGTTG |  | +48 |  |
| **24***-****LkGDH1*** | CAAGCATCTGTAGATACGCAC | +25 | -31 | 111 |
| **24***-****LkGDH1*** | TGGAACAAAGTAGAGTCCTTC |  | +80 |  |
| **25***-****LkGDH1*** | CAACAAGCTTACAACGAAGTC | +83 | +28 | 109 |
| **25***-****LkGDH1*** | ATGATTCTTTCCGGAACAGAGA |  | +137 |  |
| **26***-****LkGDH1*** | GAAGGACTCTACTTTGTTCCA | +112 | +60 | 103 |
| **26***-****LkGDH1*** | CATTTTCCCAAGTGACTCTGAAT |  | +163 |  |
| **27***-****LkGDH1*** | TCTCTGTTCCGGAAAGAATCAT | +168 | +116 | 103 |
| **27***-****LkGDH1*** | CTTGGCAGAGTTGTATTGAAC |  | +219 |  |

**D)**

| **Primer** | **Sequence** | **Middle of amplicon (Promoter coordinate)** | **5’/3’ end** | **Size (bp)** |
| --- | --- | --- | --- | --- |
| **1*-KlGDH1*** | ATCGCGGCCATTTCCACCA | -880 | -934 | 108 |
| **1*-KlGDH1*** | ACTCGACCGCTTCTAAATTGT |  | -826 |  |
| **2*-KlGDH1*** | AGGAAATGTGCTAACTGATTGT | -851 | -906 | 111 |
| **2*-KlGDH1*** | AACTAAGGCAGTCTAAGCGTTT |  | -795 |  |
| **3*-KlGDH1*** | ACAATTTAGAAGCGGTCGAGT | -802 | -846 | 89 |
| **3*-KlGDH1*** | TCCTAGAAGTAACCGTGGAAT |  | -757 |  |
| **4*-KlGDH1*** | AAACGCTTAGACTGCCTTAGTT | -767 | -816 | 98 |
| **4*-KlGDH1*** | AACCCTTCCGTTAAAAGTATCAT |  | -718 |  |
| **5*-KlGDH1*** | ATTCCACGGTTACTTCTAGGA | -723 | -777 | 109 |
| **5*-KlGDH1*** | TTGCCAGTACTATTCCCAGAA |  | -668 |  |
| **6*-KlGDH1*** | ATGATACTTTTAACGGAAGGGTT | -679 | -740 | 123 |
| **6*-KlGDH1*** | TCGTTTTCAGCTCATATTGGCAT |  | -617 |  |
| **7*-KlGDH1*** | TTCTGGGAATAGTACTGGCAA | -635 | -689 | 108 |
| **7*-KlGDH1*** | GCTAAGATAAGGAAATTCGTGAT |  | -581 |  |
| **8*-KlGDH1*** | ATGCCAATATGAGCTGAAAACGA | -598 | -639 | 82 |
| **8*-KlGDH1*** | GCTCTGTTCAAATACTTTCTTTTC |  | -557 |  |
| **9*-KlGDH1*** | ATCACGAATTTCCTTATCTTAGC | 548 | -603 | 111 |
| **9*-KlGDH1*** | CACTTTCGCACTTTCCGATC |  | -492 |  |
| **10*-KlGDH1*** | GAAAAGAAAGTATTTGAACAGAGC | -519 | -580 | 113 |
| **10*-KlGDH1*** | CTGGGCAATAGAATAACTCAAAG |  | -467 |  |
| **11*-KlGDH1*** | GATCGGAAAGTGCGAAAGTG | -465 | -511 | 93 |
| **11*-KlGDH1*** | CATCCCATTCAAACAAGAGTTAC |  | -418 |  |
| **12*-KlGDH1*** | CTTTGAGTTATTCTATTGCCCAG | -440 | -489 | 98 |
| **12*-KlGDH1*** | CTTTGAGTTATTCTATTGCCCAG |  | -391 |  |
| **13*-KlGDH1*** | GTAACTCTTGTTTGAATGGGATG | -392 | -440 | 97 |
| **13*-KlGDH1*** | GGACCAATTTTTTCTTTTGCGAT |  | -343 |  |
| **14*-KlGDH1*** | GCATTATTTCAGATGAAGAAGAAG | -371 | -414 | 86 |
| **14*-KlGDH1*** | TCAATCACCAATCAAATACAGGA |  | -328 |  |
| **15*-KlGDH1*** | ATCGCAAAAGAAAAAATTGGTCC | -317 | -365 | 95 |
| **15*-KlGDH1*** | ATAGCCACAAATTACAAAGAGGA |  | -270 |  |
| **16*-KlGDH1*** | TCCTGTATTTGATTGGTGATTGA | -290 | -340 | 99 |
| **16*-KlGDH1*** | TTAAAACTGTATAACTGCCAGAC |  | -241 |  |
| **17*-KlGDH1*** | TCCTCTTTGTAATTTGTGGCTAT | -246 | -292 | 93 |
| **17*-KlGDH1*** | TGGAACAAGTCAACTAACTACC |  | -199 |  |
| **18*-KlGDH1*** | GTCTGGCAGTTATACAGTTTTAA | -211 | -263 | 104 |
| **18*-KlGDH1*** | GAGGCAAGAGATGTAAACCAT |  | -159 |  |
| **19*-KlGDH1*** | GGTAGTTAGTTGACTTGTTCCA | -171 | -220 | 98 |
| **19*-KlGDH1*** | GCACTGTAATCAAGAAGTAAAAAG |  | -122 |  |
| **20*-KlGDH1*** | ATGGTTTACATCTCTTGCCTC | -126 | -179 | 107 |
| **20*-KlGDH1*** | CGTACTGTATCGTAGCCTAAG |  | -72 |  |
| **21*-KlGDH1*** | CTTTTTACTTCTTGATTACAGTGC | -98 | -145 | 94 |
| **21*-KlGDH1*** | CAATCTATGATGAAAGTAATTCGTA |  | -51 |  |
| **22*-KlGDH1*** | CTTAGGCTACGATACAGTACG | -38 | -92 | 109 |
| **22*-KlGDH1*** | TATGGTTCAGCAGACATCTTTAA |  | +17 |  |
| **23*-KlGDH1*** | TACGAATTACTTTCATCATAGATTG | +18 | -75 | 115 |
| **23*-KlGDH1*** | AGGCTTGTTGGAATTCTGGTT |  | +40 |  |
| **24*-KlGDH1*** | TTAAAGATGTCTGCTGAACCATA | +43 | -6 | 97 |
| **24*-KlGDH1*** | GTTTCTTGTCGAACAAAGTAGAG |  | +91 |  |
| **25*-KlGDH1*** | AACCAGAATTCCAACAAGCCT | +73 | +20 | 105 |
| **25*-KlGDH1*** | CAGAGACAACTGGCAAAACCT |  | +125 |  |
| **26*-KlGDH1*** | CTCTACTTTGTTCGACAAGAAAC | +121 | +69 | 103 |
| **26*-KlGDH1*** | CTTTGTCATTTTCCCAGGTAAC |  | +172 |  |
| **27*-KlGDH1*** | AGGTTTTGCCAGTTGTCTCTG | +154 | +104 | 99 |
| **27*-KlGDH1*** | ACTCTGAAACCAGTAGCGACT |  | +203 |  |
| **28*-KlGDH1*** | GTTACCTGGGAAAATGACAAAG | +202 | +151 | 99 |
| **28*-KlGDH1*** | TGGAATCTCAAACCACCCTTG |  | +251 |  |
